# Acylhydrazones and Their Biological Activity: A Review

**DOI:** 10.3390/molecules27248719

**Published:** 2022-12-09

**Authors:** Laura-Ileana Socea, Stefania-Felicia Barbuceanu, Elena Mihaela Pahontu, Alexandru-Claudiu Dumitru, George Mihai Nitulescu, Roxana Corina Sfetea, Theodora-Venera Apostol

**Affiliations:** 1 Faculty of Pharmacy, “Carol Davila” University of Medicine and Pharmacy, 6 Traian Vuia Street, District 2, 020956 Bucharest, Romania; 2 Faculty of Medicine, “Carol Davila” University of Medicine and Pharmacy, 8 Eroii Sanitari Boulevard, District 5, 050474 Bucharest, Romania

**Keywords:** acylhydrazone, intermediates, synthesis, properties, cytotoxic, antimicrobial, antiviral, antioxidant, anti-inflammatory, antiparasitic

## Abstract

Due to the structure of acylhydrazones both by the pharmacophore –CO–NH–N= group and by the different substituents present in the molecules of compounds of this class, various pharmacological activities were reported, including antitumor, antimicrobial, antiviral, antiparasitic, anti-inflammatory, immunomodulatory, antiedematous, antiglaucomatous, antidiabetic, antioxidant, and actions on the central nervous system and on the cardiovascular system. This fragment is found in the structure of several drugs used in the therapy of some diseases that are at the top of public health problems, like microbial infections and cardiovascular diseases. Moreover, the acylhydrazone moiety is present in the structure of some compounds with possible applications in the treatment of other different pathologies, such as schizophrenia, Parkinson’s disease, Alzheimer’s disease, and Huntington’s disease. Considering these aspects, we consider that a study of the literature data regarding the structural and biological properties of these compounds is useful.

## 1. Introduction

**Figure 1:**
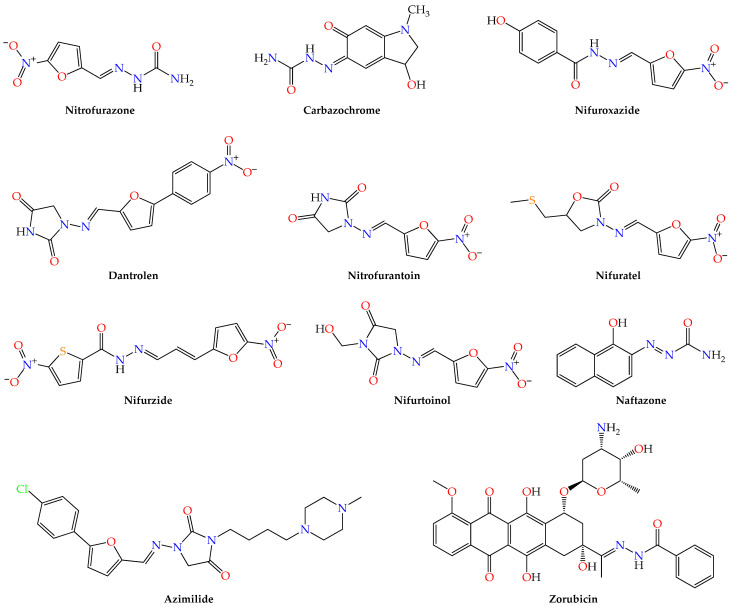
Structures of some representative bioactive molecules bearing the acylhydrazone template.

The acylhydrazones, through their structure, have significant malleability both chemically and pharmaceutically. Numerous representatives of this class of organic compounds are intermediates in the synthesis of heterocyclic compounds, including pentatomic ones [1,2,3,4,5,6]. They also present a structural variability that offers the possibility to synthesize compounds belonging to this class with various therapeutic indications (like cytotoxic, antibacterial, antifungal, antiviral, antioxidant, antiparasitic, anti-inflammatory, anticonvulsant, and antihypertensive) [7,8]. A number of derivatives containing the acylhydrazone moiety are used in therapy, such as nitrofurazone (antimicrobial), carbazochrome (antihemorrhagic), nifuroxazide (intestinal antibacterial), dantrolene (muscle relaxant), nitrofurantoin (antibacterial), nifuratel (antitrichomonal and antifungal), nifurzide (intestinal anti-infective), nifurtoinol (urinary anti-infective), naftazone (capillary stabilizing), azimilide (anti-arrhythmic), zorubicin (cytotoxic antibiotic) [9,10,11]. The structures of some representative pharmacologically active agents containing the acylhydrazone scaffold are shown in Figure 1.

The objective of this paper is to review the literature describing the acylhydrazone moiety as an important scaffold for medicinal chemistry highlighting its versatility and drug-like character.

## 2. Structure

The acylhydrazones have in their structure the –CO–NH–N=CH– group in which there are: an electrophilic carbon atom (CH=N), a nucleophilic imine nitrogen atom, by the doublet of non-participating electrons (CH=N:), and an amino nitrogen atom with acidic character (–NH–) [12,13]. Thus, the acylhydrazone molecules are both electrophilic and nucleophilic [14]. The nucleophilic attack is performed at the amine nitrogen atom (NH), and the electrophilic one at the oxygen atom (CO) [15].

The acylhydrazones can also exhibit keto-enol tautomerism and through the electron donor (the oxygen atom of the carbonyl group) [14], together with the azomethine nitrogen atom (–N=), participate in the chelation of metal ions [16].

Due to the fact that the N=CH bond is in the vicinity of the amide nitrogen atom (CO–NH), the acylhydrazones may have an acidic character manifested by the yielding of the hydrogen atom bound to the azomethine carbon atom [17].

The acylhydrazones can form intermolecular hydrogen bonds through the hydrogen atom bound to the amino nitrogen (–NH–) and the oxygen atom [18,19,20], between the hydrogen atom bound to the imine carbon (CH) and the atomic nitrogen atom (–N=) of another molecule [20].

The acylhydrazones exhibit geometric isomerism due to the imine group (–N=CH–). Thus, they are in a mixture of *E* and *Z* isomers, where *E* is predominant, in general, because its stability is superior to the *Z* isomer [4,21].

**Figure 2:**
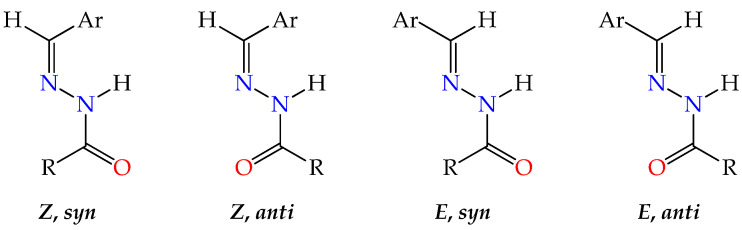
Isomers of acylhydrazone derivatives.

Theoretically, the acylhydrazones can have four isomers, two of which are geometric isomers (*E*/*Z*) and are due to the C=N double bond, and two are conformal isomers (syn/anti) and are due to the N–N bond [5,22]. The structures of these isomers are shown in Figure 2 [14].

**Figure 3:**
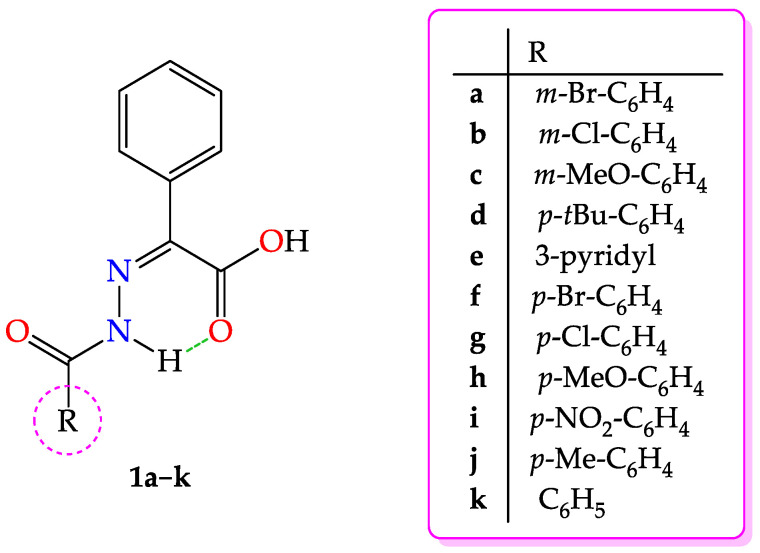
Intramolecular hydrogen bond.

In the case of *N*-aroylhydrazones **1a**–**k** (Figure 3), the *Z* isomer is stabilized by intramolecular hydrogen bonds. Thus, it is found in a higher percentage than the *E* isomer [5].

The NMR spectra indicated that the *N*-acylhydrazones usually exist as a mixture of two conformers, namely *E*_(C=N)(N–N)_ *synperiplanar* and *E*_(C=N)(N–N)_ *antiperiplanar*, at room temperature in DMSO-*d*_6_. The *E*_(C=N)_ configurational isomers rapidly establish *synperiplanar*/*antiperiplanar* equilibrium about the –CO–NH– bond, in the DMSO-*d*_6_ solution. The *synperiplanar* conformer predominates the *antiperiplanar* isomer due to its ability to develop intermolecular interactions with polar solvents, like DMSO [23].

## 3. Synthesis

**Scheme 1:**
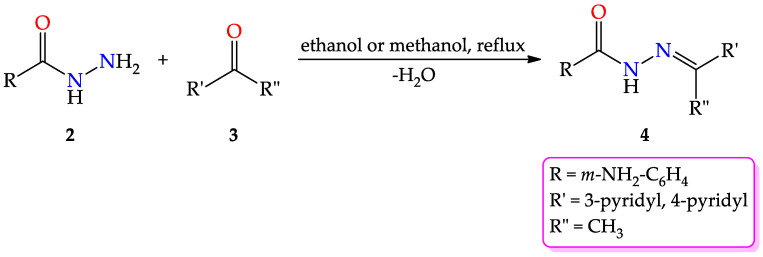
General synthesis reaction of acylhydrazones.

The acylhydrazones **4** can be obtained by the condensation reaction of an aldehyde or ketone **3** with a derivative of the class of hydrazides **2** [24] in the presence of an alcohol [25,26], generally at reflux, and in an acidic medium [12,27,28,29,30,31,32] or in the absence of the acid catalyst [4,6,33,34,35,36,37]. The general synthesis reaction of acylhydrazones is presented in Scheme 1 [37].

## 4. Spectral Analysis

The vibration-rotation spectra of acylhydrazones show bands specific to the –CO–NH–N= moiety present in the structure of derivatives of this class. The intervals in which these bands are recorded are as follows: 1647–1687 cm^−1^ for the C=O connections [6,18,19], 3194–3440 cm^−1^ for the NH connection [6,19,29], with the specification that there is variation between symmetrical (3080 cm^−1^) and asymmetrical vibrations (3194 cm^−1^) [17], 980–1000 cm^−1^ for the N–N connection [38], 1578–1623 cm^−1^ for the N=C connection [17,18,19], and for the CH connection the value of the wavenumber in the region of 3050–3078 cm^−1^ was reported [6].

The values of the chemical shifts of the protons specific to the acylhydrazone derivatives in the ^1^H-NMR spectra are in the following ranges: 11.0–13.5 ppm for the proton of the –CO–NH– group [6,31], 8.5–12.5 ppm for the proton of the N–H bond [12,19,28], 8–9 ppm for the proton of the –N=CH– group [12,31].

In the ^13^C-NMR spectra, the chemical shift values for the imine carbon atom (–N=CH–) are between 157–168 ppm, and for the amide carbon atom (–CO–NH–) are reported between 159.0–173.5 ppm [6,31]. In some cases, the duplicated signals observed in the NMR spectra of acylhydrazones correspond to the presence of two amide bond-related conformers [23].

## 5. Biological Properties

Acylhydrazones have significant importance in the pharmaceutical field through numerous biological properties with multiple therapeutic indications. In the research studies performed using compounds from the acylhydrazone class, the following actions were reported: antitumor [25,26,27,28,39,40,41] and [42,43,44,45,46,47,48,49,50,51], cytotoxic [52], antibacterial [4,12,31,33,34,35] [53,54,55,56,57,58,59], antifungal [34,60,61], antiviral [62,63,64,65,66,67,68,69], antiparasitic [6,45,70,71,72,73,74,75,76], anti-inflammatory [32,73,77,78,79,80,81,82,83,84], analgesic [36,77,78,79,80,81,85], immunomodulatory [83,86], enzyme inhibition [29,86,87,88] and [89,90,91,92,93,94,95,96,97,98], antidiabetic [18], anticonvulsant [73], antioxidant [34,39,78,99,100,101], and effects on the cardiovascular system [102,103,104,105,106,107,108,109,110,111].

### 5.1. Antitumor Action

**Figure 4:**
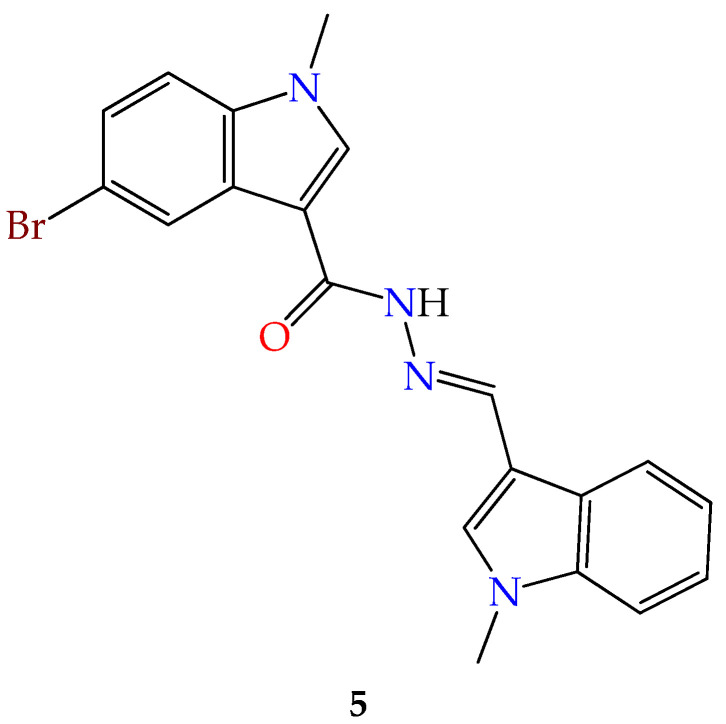
Structure of 5-bromo-1-methyl-*N*’-[(*E*)-(1-methyl-1*H*-indol-3-yl)methylidene]-1*H*-indole-3-carbohydrazide **5** with antitumor action.

According to a recent study, 5-bromo-1-methyl-*N*’-[(*E*)-(1-methyl-1*H*-indol-3-yl)methylidene]-1*H*-indol-3-carbohydrazide **5** (Figure 4) showed antitumor action on breast, cervical and colon cancer cell lines by inducing cellular apoptosis. This action is exerted through cyclic adenosine monophosphate (cAMP)-dependent protein kinase A, p53 protein, and by stimulating the generation of reactive oxygen species (ROS) and nitric oxide (NO) [28].

**Figure 5:**
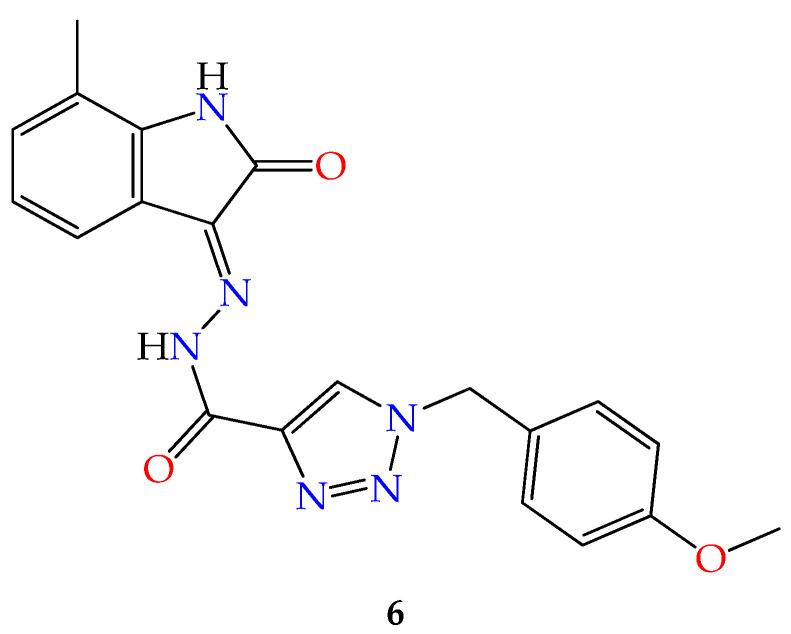
Structure of (*E*)-1-(4-methoxybenzyl)-*N*’-(7-methyl-2-oxoindolin-3-ylidene)-1*H*-1,2,3-triazole-4-carbohydrazide **6** with antiproliferative action.

Aneja et al. demonstrated that (*E*)-1-(4-methoxybenzyl)-*N*’-(7-methyl-2-oxoindolin-3-ylidene)-1*H*-1,2,3-triazole-4-carbohydrazide **6** (Figure 5) has an inhibitory effect on the kinase involved in cell replication, microtubule affinity regulatory kinase (4MARK4), fulfilling an antiproliferative effect simultaneously with increasing the production of ROS, inducing apoptosis in cancer cell lines [25].

**Figure 6:**
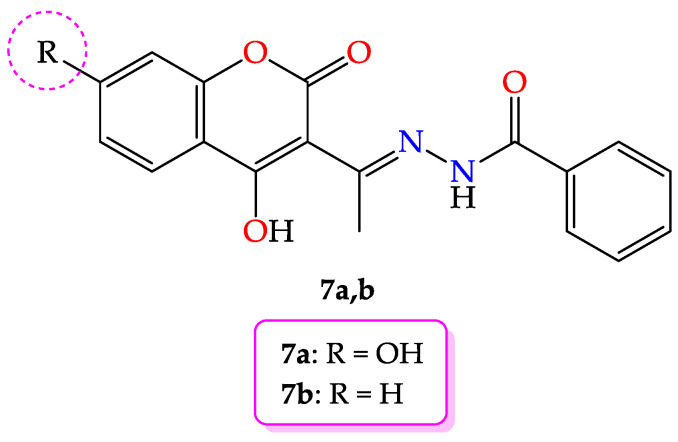
Structures of *N*’-(1-(4,7-dihydroxy-2-oxo-2*H*-chromen-3-yl)ethylidene)benzohydrazide **7a** and *N*’-(1-(4-hydroxy-2-oxo)-2*H*-chromen-3-yl)ethylidene)benzohydrazide **7b** with cytotoxic action.

The cytotoxic action was evidenced for several compounds of which two derivatives, namely *N*’-(1-(4,7-dihydroxy-2-oxo-2*H*-chromen-3-yl)ethylidene)benzohydrazide **7a** and *N*’-(1-(4-hydroxy-2-oxo-2*H*-chromen-3-yl)ethylidene)benzohydrazide **7b** (Figure 6), were identified as having an intensity of this effect comparable to that of doxorubicin and colchicine [27].

**Figure 7:**
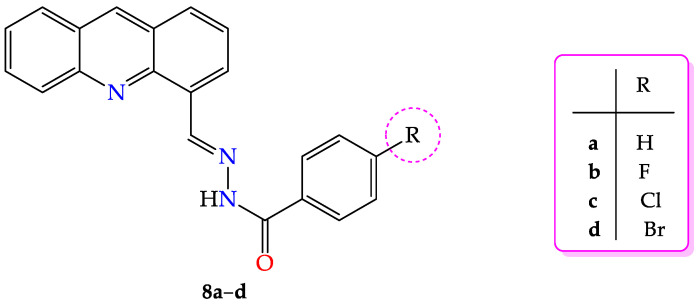
Structures of acridine-benzohydrazides **8a**–**d** with anticancer activity.

Very recently, Vilková et al. investigated the anticancer activity of some acridine acylhydrazone analogs **8a**–**d** (Figure 7), among which **8a** and **8c** reduced the clonogenic capacity of A549 cells [112].

**Figure 8:**
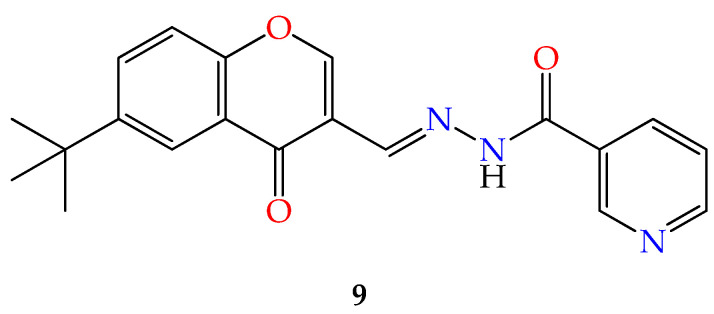
Structure of compound **9** showing antitumor action.

According to the evaluation of Lis et al., acylhydrazone **9** (Figure 8) induced apoptosis in erlotinib-resistant neoplasms as a result of selective STAT3 inhibition [40].

**Figure 9:**
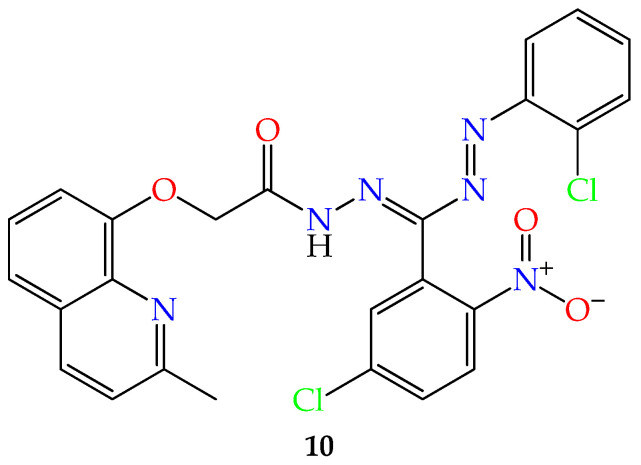
Structure of azo-hydrazone analog **10** with antiproliferative activity.

Recently, Banumathi et al. showed that the azo-hydrazone analog **10** (Figure 9) exerted chemosensitivity specifically against EAC and A549 cells without altering their normal counterpart [113]. It was found that the antiproliferative activity of **10** was due to the induction of apoptosis by inhibiting the STAT3 signal. Furthermore, compound **10** attenuated solid tumor growth without inducing significant toxicological side effects.

**Figure 10:**
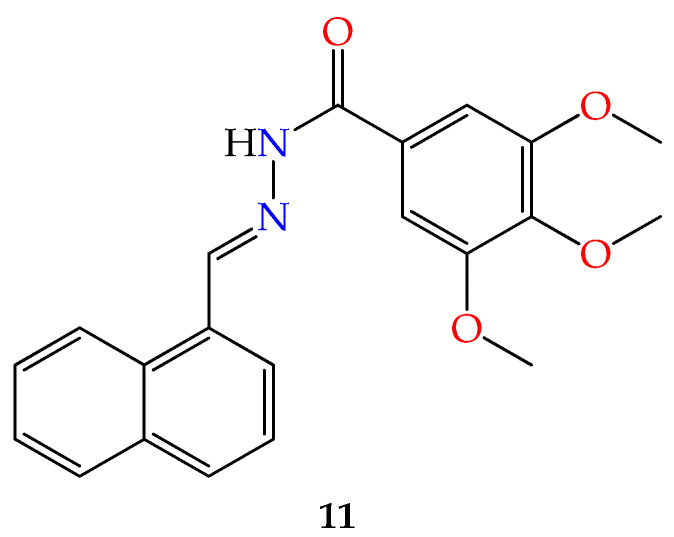
Structure of derivative **11** with antiproliferative action.

The acylhydrazone derivative **11** (Figure 10) exhibited an in vivo antiproliferative effect with a potency similar to that of colchicine both by inducing apoptosis and by inhibiting the polymerization of microtubules [26].

**Figure 11:**
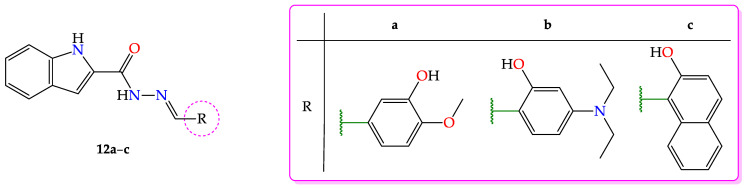
Structures of indole-hydrazone derivatives **12a**–**c** with antiproliferative and antioxidant actions.

The derivatives **12a**–**c** (Figure 11) presented, besides the antiproliferative action on human erythroleukemia K562 and melanoma Colo-38 cells, an antioxidant action demonstrated based on 1,1-diphenyl-2-picrylhydrazyl (DPPH) radical scavenging activity test, ferric reducing antioxidant power, and oxygen radical absorbance capacity [39].

**Figure 12:**
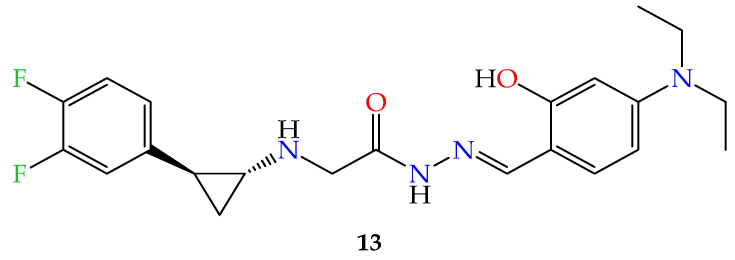
Structure of acylhydrazone **13** with antitumor action.

According to a study by Sun et al., it was found that a derivative of the class of acylhydrazones (**13**) (Figure 12) showed antitumor action with possible use in gastric cancer as a lysine-specific demethylase 1 (LSD1) inhibitor [41].

**Figure 13:**
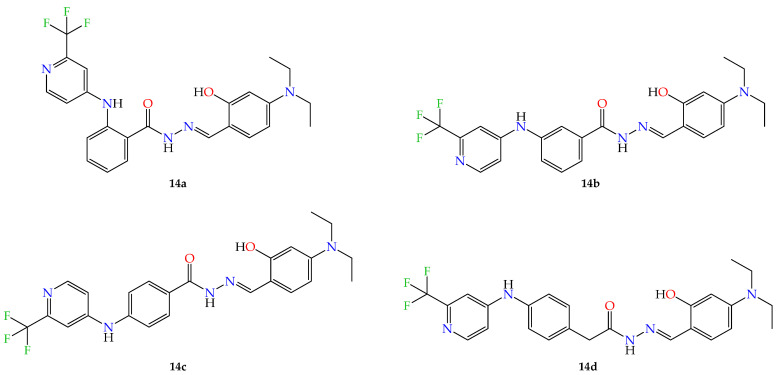
Structures of the acylhydrazone class compounds **14a**–**d** with cytotoxic effect.

Congiu et al. synthesized a series of acylhydrazone derivatives **14a**–**d** (Figure 13) which showed cytotoxic effect and inhibition of tumor development for a relatively large number of neoplasms [42].

**Figure 14:**
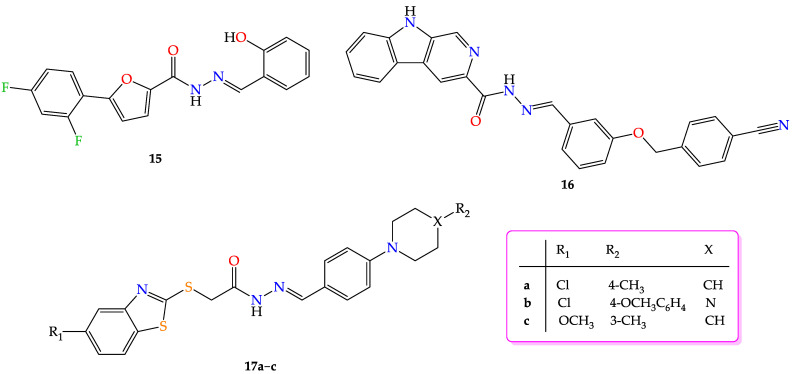
Structures of acylhydrazones **15**–**17** with cytotoxic action.

A series of acylhydrazone-derived compounds displayed cytotoxic action of variable intensity. Thus, for compound **15**, the potency of the effect was higher compared to doxorubicin in promyelocytic leukemia [43]; for the acylhydrazone derivative **16**, the intensity of the effect was significant due to the exercise of cytotoxic action on different neoplasms including resistant cell lines [44]. The benzothiazole acylhydrazones **17a**–**c** showed selective inhibition towards cancer cells. Moreover, derivative **17a** displayed higher antiproliferative activity than the reference agent cisplatin [114]. The structures of the acylhydrazones **15**–**17** are presented in Figure 14.

**Figure 15:**
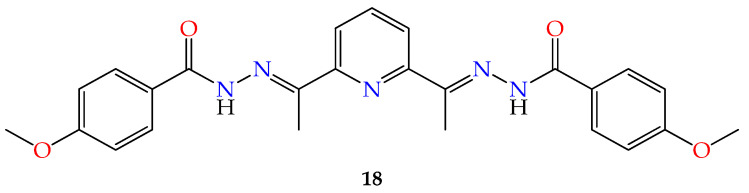
Structure of the acylhydrazone derivative **18** with cytotoxic action.

In the case of acylhydrazone derivatives, the cytotoxic mechanism does not involve the generation of ROS leading to apoptosis. The derivative **18** (Figure 15) falls into this category of compounds, influencing the cell cycle, cell division, and ribonucleotide reductase, an enzyme that changes its activity following the chelation of iron ions [46].

**Figure 16:**
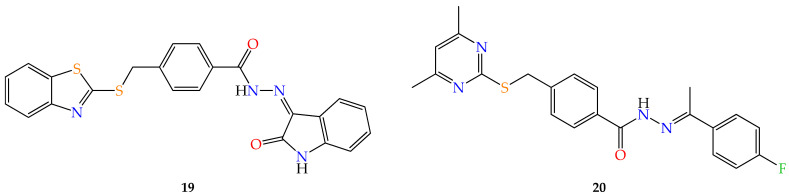
Structures of compounds **19** and **20** with cytotoxic action.

In a study by Yu et al., two derivatives of the class of acylhydrazones **19** and **20** (Figure 16) with cytotoxic action superior to the reference substance (5-fluorouracil) were reported [47].

**Figure 17:**
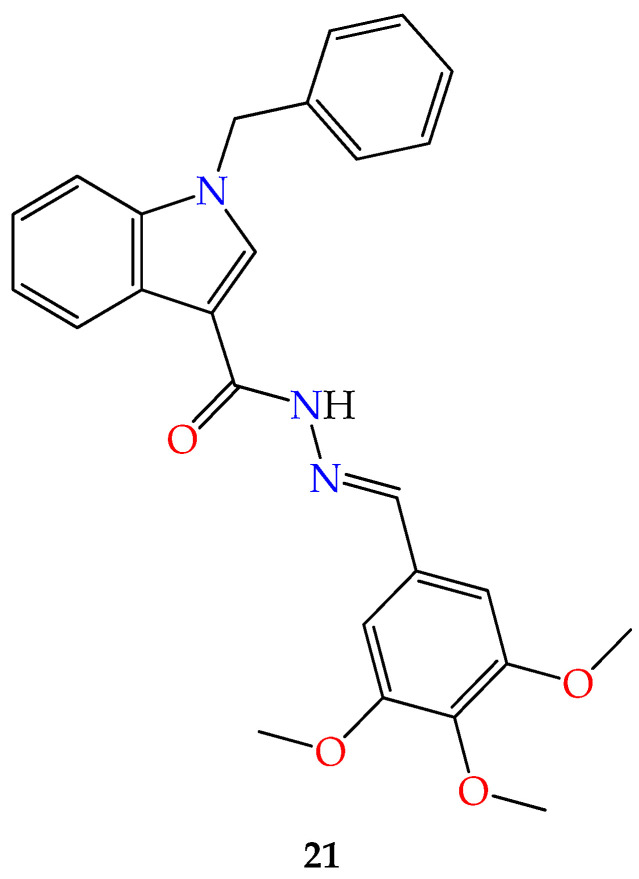
Structure of acylhydrazone derivative **21** with antitumor action.

Acylhydrazone **21** (Figure 17) could be used in therapy as an antitumor agent with insignificant effects on normal cell lines due to the fact that it induces apoptosis by depolarizing the mitochondrial membrane and generating ROS in cancer cell lines. In addition to these actions, the compound is involved in the inhibition of tubulin polymerization [48].

**Figure 18:**
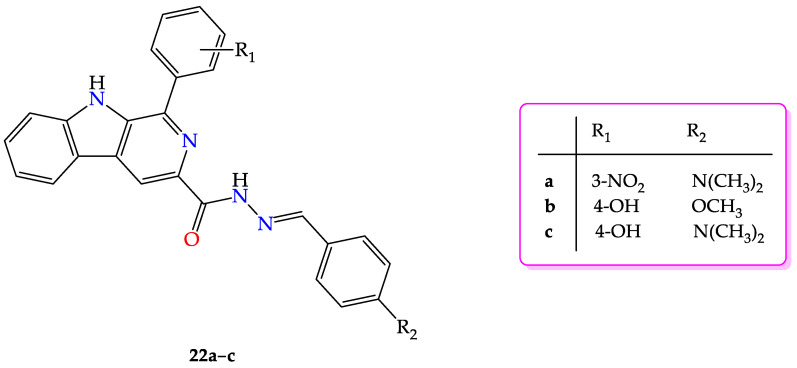
Structures of acylhydrazones **22a**–**c** with cytotoxic action.

Compound **22a** showed the strongest cytotoxic action on all cell cultures used, and derivatives **22b** and **22c** exhibited cytotoxicity only on a certain (ovarian cancer) cell line. In the experimental model of Ehrlich solid carcinoma, the acylhydrazone **22a** showed inhibition of tumor development comparable to that of the reference substance, 5-fluorouracil [49]. The structures of acylhydrazones **22a**–**c** are presented in Figure 18.

**Figure 19:**
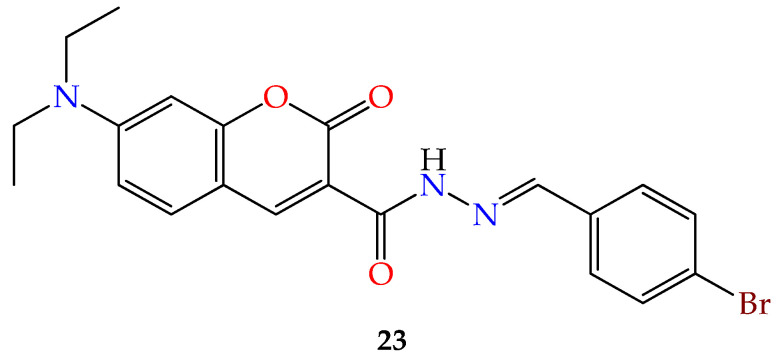
Structure of compound **23** with intense antitumor action.

De Almeida et al. evaluated the cytotoxic action of a series of derivatives from the class of acylhydrazones. The research showed that acylhydrazone **23** (Figure 19) exerted the best action in the series of studied compounds, probably due to the bromine substituent in the *para* position on the phenyl nucleus [50].

**Figure 20:**
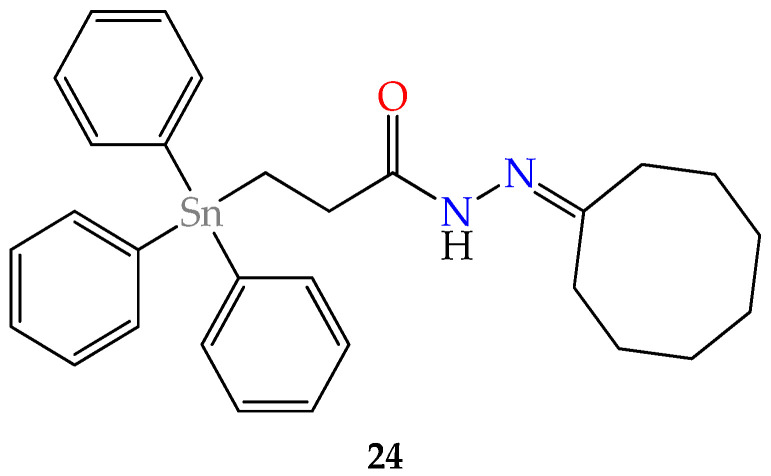
Structure of the acylhydrazone derivative **24** with antitumor action.

The acylhydrazone derivative **24** (Figure 20) showed antitumor action on the studied cancer cell lines with increased intensity on the lung cancer cell line. The cytotoxic action is due to the generation of ROS and the altering of the cell cycle [51].

**Figure 21:**
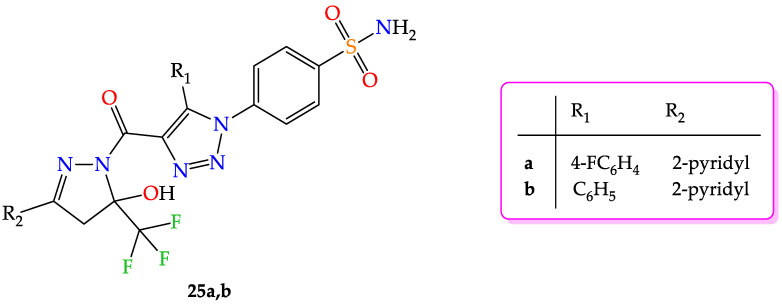
Structures of acylhydrazone compounds **25a**,**b** with antitumor effect.

Compounds **25a** and **25b** (Figure 21) showed the inhibitory effect against carbonic anhydrase IX and XII isoforms, respectively, involved in the growth and development of tumors [29].

**Figure 22:**
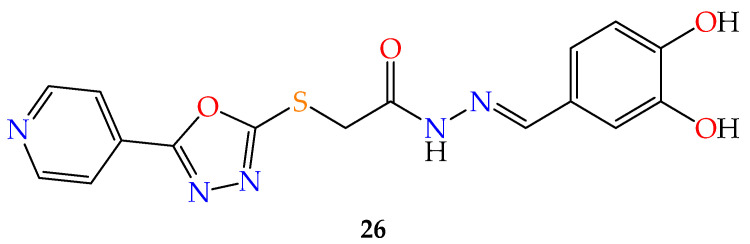
Structure of acylhydrazone **26** with antitumor action.

The derivative **26** (Figure 22) showed antitumor action with a higher potency compared to 5-fluorouracil, due to the inhibitory effect of telomerase [91].

**Figure 23:**
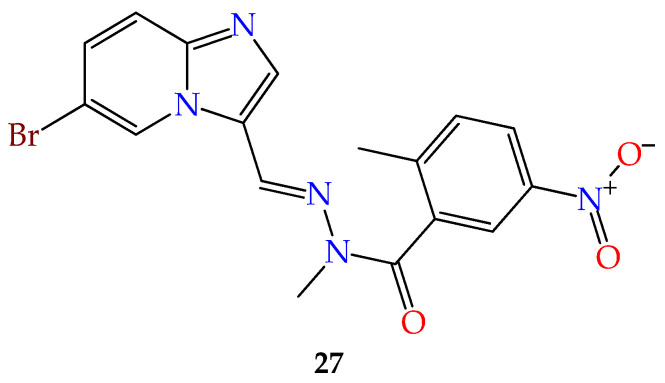
Structure of compound **27** with antitumor activity.

The acylhydrazone **27** (Figure 23) demonstrated the inhibitory activity on phosphatidyl-inositol-3-kinase, which is involved in cell division. Gao et al. assumed that the action was feasible due to the nitrogen atoms and substituents in the compound structure [94].

**Figure 24:**
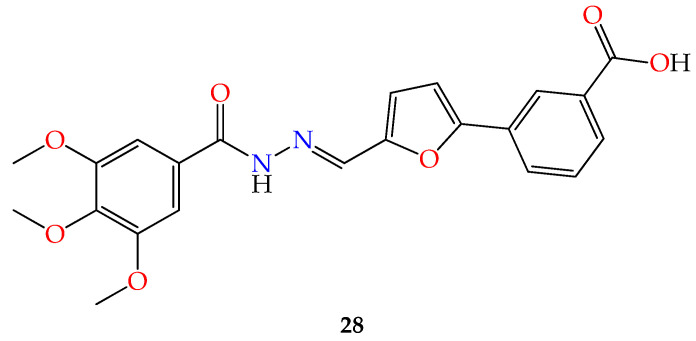
Structure of compound **28** with lactate dehydrogenase A inhibitory action.

The acylhydrazone **28** (Figure 24) was reported as an inhibitor with significant action on lactate dehydrogenase A, an isoform that exhibits abnormal activity in tumor cells [96].

### 5.2. Antimicrobial Action

#### 5.2.1. Antibacterial Action

There are many acylhydrazones described to have antimicrobial effects on various bacterial strains. It is difficult to analyze the structure-activity relationships because of the high chemical diversity of these compounds. As a general rule, the compounds active on Gram-negative bacteria are more hydrophilic than those effective on Gram-positive bacteria because of the differences in their cell wall structure [115,116]. Many of the studies reported here used acylhydrazone scaffold as the rationale for their drug-design process and presented only the phenotypic antibacterial activity without the mechanism of the effect.

**Figure 25:**
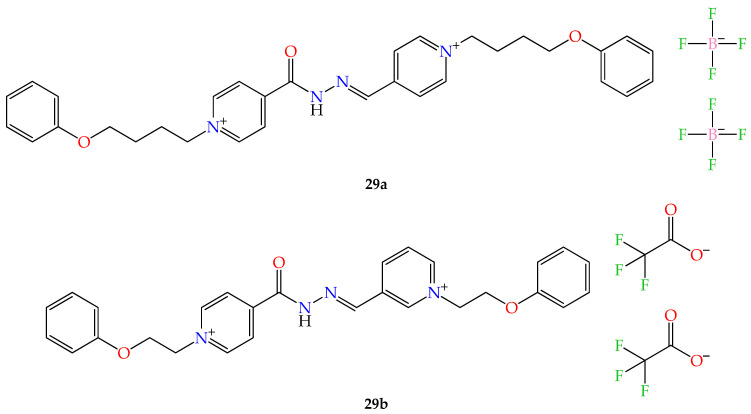
Structures of the salts of acylhydrazones **29a**,**b** with antimicrobial action.

A series of acylhydrazone salts **29a**,**b** (Figure 25) were synthesized and their antimicrobial action was studied. It is noteworthy that the investigated derivative **29a** exerted antimicrobial action on methicillin-resistant *Staphylococcus aureus* (MRSA), *Escherichia coli*, *Clostridium difficile*, and *Candida albicans*. A high potency action was registered on methicillin-resistant *Staphylococcus aureus* and *Escherichia coli* (**29b**) [31]. Overall, molecular dynamics simulation analysis showed that the effect of structural features, such as pyridinium scaffold, hydrophobic side chains, and –CO–NH–N= linker, in the diffusion of such substances across the cell membrane and that it could be responsible for their antibacterial activity. In order to understand the mechanism of acylhydrazone salts **29a**,**b** as anti-bacterial agents, docking experiments were performed against the microbial target, *E. coli* glucosamine-6-P synthase. The acylhydrazone salts **29a**,**b** were predicted to form stable hydrogen bonding and hydrophobic interactions. Molecular dynamics simulation highlighted the target interaction behavior of these derivatives at the surface of cell membranes indicating a passive diffusion mechanism at the surface layer.

**Figure 26:**
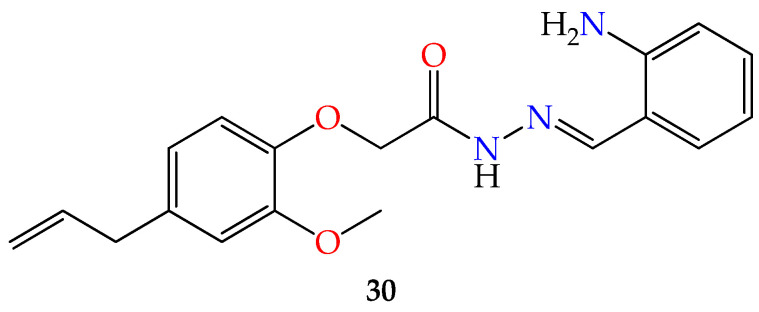
Structure of acylhydrazone **30** with tuberculostatic action.

Among the pathogenic microorganisms, for which the antimicrobial action of acylhydrazone derivatives was demonstrated there is also *Mycobacterium tuberculosis*. Rohane et al. synthesized an acylhydrazone **30** (Figure 26) with the most intense action among the obtained derivatives due to the substituents on the benzene ring. The reference substance used was isoniazid [33]. Molecular docking studies investigating acylhydrazone analogs using enoyl acyl carrier protein reductase as their potential biological target indicate that the hydroxyl, azide, amino, and phenyl groups of the spacer of the acylhydrazone play an important role in the interactions with the active site [33]. The enoyl acyl carrier protein reductase is an attractive target for drug-design, being essential in the type II fatty acid synthase system found in microorganisms and without homologue in mammals [117].

**Figure 27:**
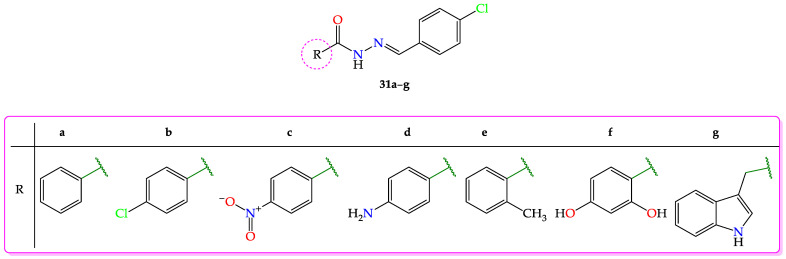
Structures of acylhydrazones **31a**–**g** with antibacterial and antifungal actions.

Siddique et al. obtained a series of new compounds **31a**–**g** (Figure 27), that showed antibacterial and antifungal actions with varying intensities studied on *Escherichia coli*, *Bacillus subtilis*, *Salmonella typhimurium*, *Staphylococcus aureus*, and *Candida albicans* [34].

**Figure 28:**
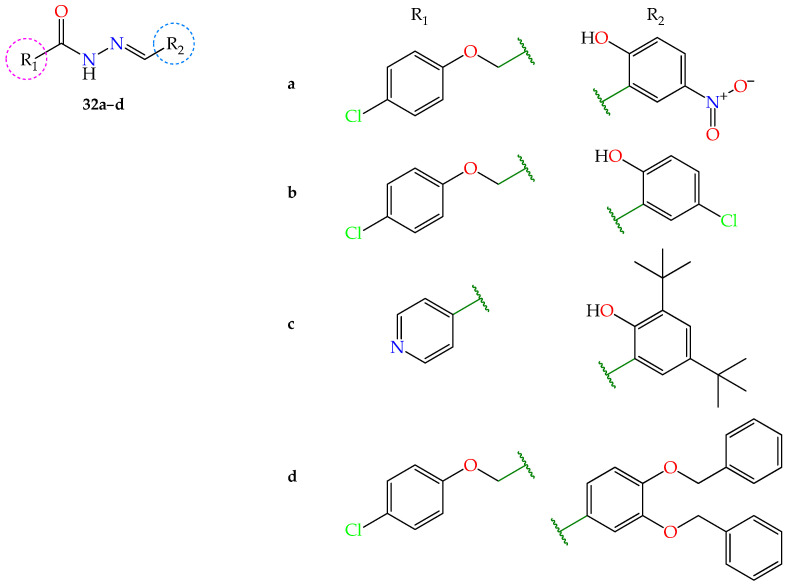
Structures of acylhydrazones **32a**–**d** with antibacterial action.

The mechanism of antibacterial action, in the case of acylhydrazones **32a**–**d** (Figure 28), studied by Xia et al., is to modulate the expression of genes responsible for hemolysis and virulence of tested pathogenic microorganisms [35].

**Figure 29:**
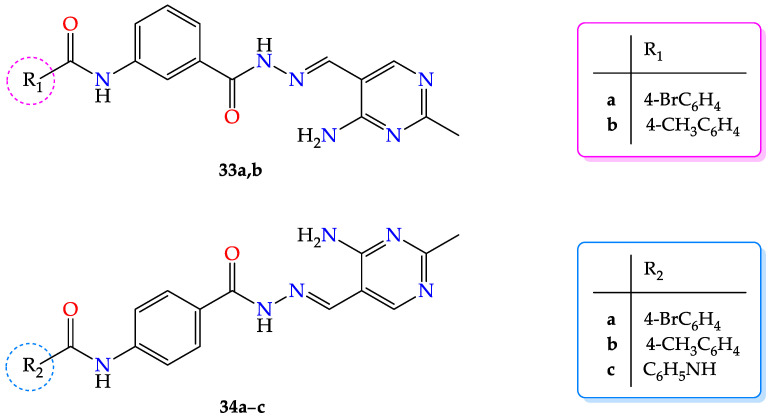
Structures of acylhydrazone derivatives **33a**,**b** and **34a**–**c** with antibacterial action.

The acylhydrazone derivatives **33a,b** and **34a**–**c** (Figure 29) showed antibacterial action on *Escherichia coli* by inhibiting the enzymatic pyruvate dehydrogenase complex (PDHc). Among the compounds studied, the most active was **34b**. The acylhydrazones **33a** and **33b** exhibited selectivity for the enzymatic complex [12].

**Figure 30:**
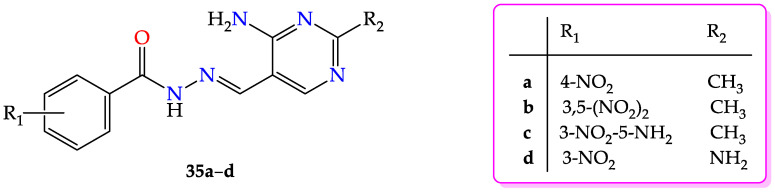
Structures of acylhydrazone derivatives **35a**–**d** with antimicrobial action.

The antimicrobial action of some acylhydrazone derivatives against *Escherichia coli*, resulting from the inhibition of the multienzyme PDHc-E1, was also investigated. Among the compounds studied, acylhydrazones **35a**–**d** (Figure 30) exerted the best action with good selectivity [53].

**Figure 31:**
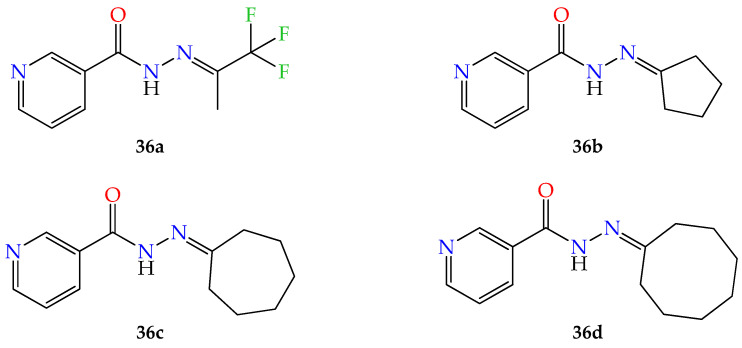
Structures of acylhydrazones **36a**–**d** with antibacterial action.

The acylhydrazone derivatives **36a**–**d** (Figure 31) showed intense antibacterial action on *Pseudomonas aeruginosa*, a resistant microorganism [4].

**Figure 32:**
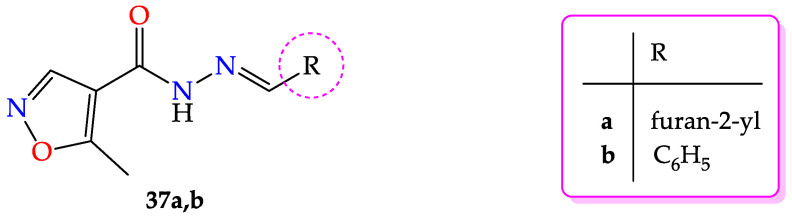
Structures of derivatives **37a,b** with antibacterial action.

According to studies by Jin et al., the acylhydrazones **37a,b** (Figure 32) exhibited a broad antibacterial spectrum, being active on both Gram-negative bacteria (*Escherichia coli*, *Pseudomonas aeruginosa*) and Gram-positive bacteria (*Bacillus subtilis*, *Staphylococcus aureus*) [54,55].

**Figure 33:**
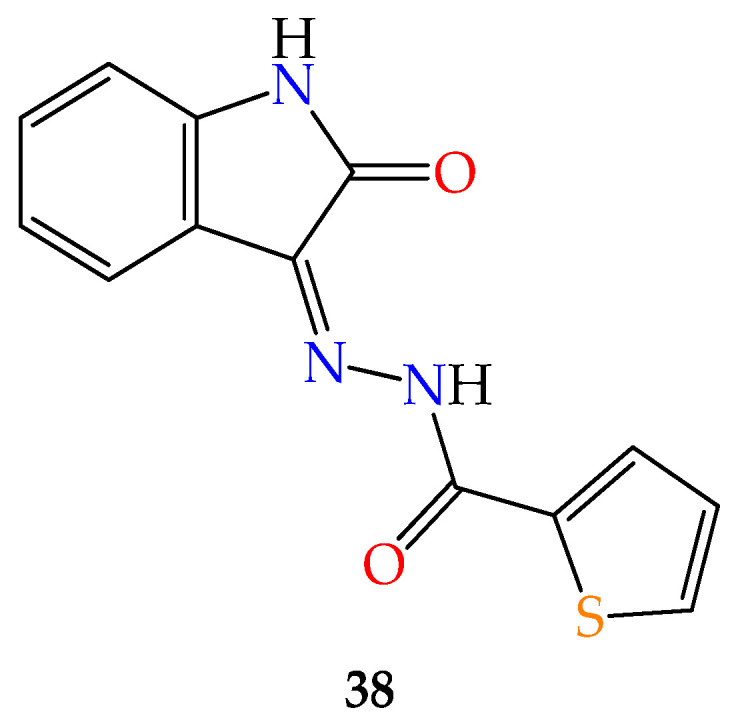
Structure of acylhydrazone **38** with antimicrobial action.

The complex of acylhydrazone **38** (H_2_L) with zinc (II) ion as [Zn(HL)_2_]∙EtOH showed an intense antimicrobial action on most of the tested bacterial strains. Among the microorganisms on which this property was studied are *Bacillus subtilis*, methicillin-resistant *Staphylococcus aureus*, *Escherichia coli*, and *Haemophilus influenzae*. The potency of the complex on *Haemophilus influenzae* was significant [56]. The structure of acylhydrazone **38** is presented in Figure 33.

**Figure 34:**
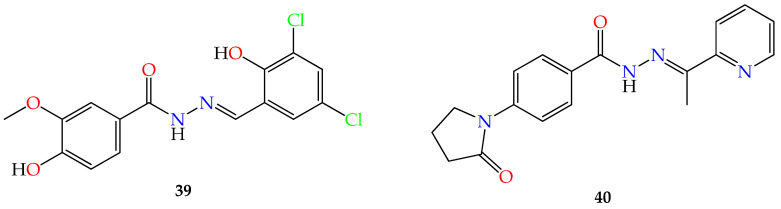
Structures of acylhydrazones **39** and **40** with antimicrobial action.

Among the compounds investigated are acylhydrazones **39** with action on *Escherichia coli*, *Pseudomonas aeruginosa*, *Staphylococcus aureus*, *Bacillus subtilis* [57] and **40** which was only active on *Mycobacterium tuberculosis* among the microorganisms included in the study [58]. The structures of acylhydrazones **39** and **40** are shown in Figure 34.

**Figure 35:**
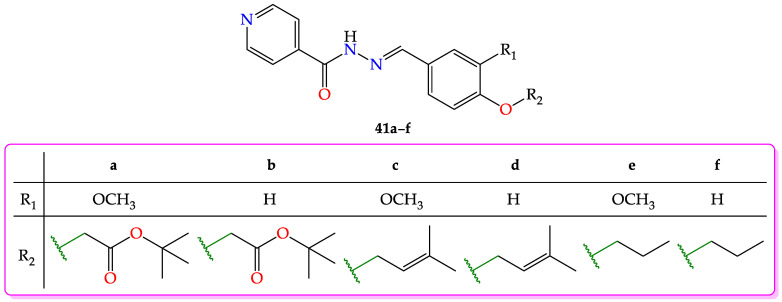
Structures of acylhydrazones **41a**–**f** with antibacterial and cytotoxic actions.

Shah et al. synthesized a series of isonicotinic hydrazid-based acylhydrazone analogs **41a**–**f** (Figure 35), which were evaluated for their antibacterial activity against two Gram-positive strains, namely *Staphylococcus aureus*, *Bacillus subtilis*, and a Gram-negative bacterium, i.e., *Escherichia coli* [118]. The results showed that the studied compounds **41a**–**f** had appreciable antibacterial activity against the tested strains, among which the derivatives **41c** and **41e** proved to be the most active, being promising agents in the treatment of bacterial infections. The acylhydrazones **41a**–**f** were also screened for their cytotoxic effect, the maximum activity being noted for analogs **41e** and **41f**.

**Figure 36:**
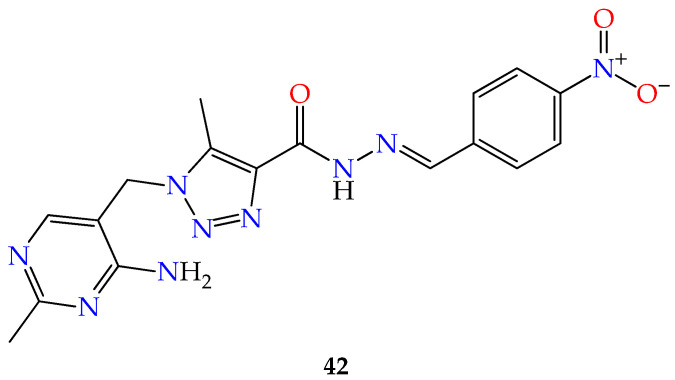
Structure of acylhydrazone **42** with antimicrobial action.

The acylhydrazone **42** (Figure 36) showed good antimicrobial activity on *Escherichia coli* by inhibiting the PDHc-E1 due to the *para*-NO_2_ group grafted on the benzene ring [93].

**Figure 37:**
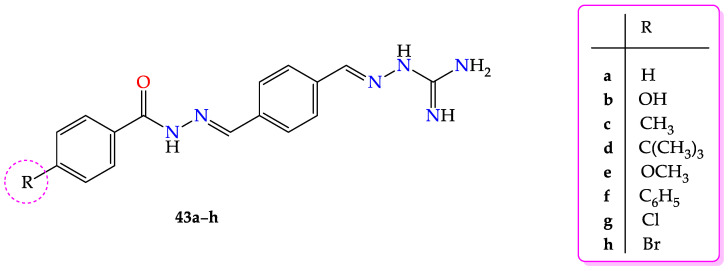
Structures of acylhydrazones **43a**–**h** screened for antibacterial activity.

Yao et al. designed and synthesized a series of aminoguanidine derivatives containing an acylhydrazone moiety **43a**–**h** (Figure 37), which were evaluated for their antimicrobial activity against Gram-positive bacteria (*Staphylococcus aureus*, *Enterococcus faecalis*, *Bacillus* s*ubtilis*, and *Streptococcus mutans*) and Gram-negative strains (*Escherichia coli*; *Pseudomonas aeruginosa*) [119]. Penicillin, oxacillin, and norfloxacin were used as positive controls. The derivative **43d** displayed a wide spectrum of antibacterial effects, being active on both Gram-positive and Gram-negative bacterial strains.

#### 5.2.2. Antifungal Action

**Figure 38:**
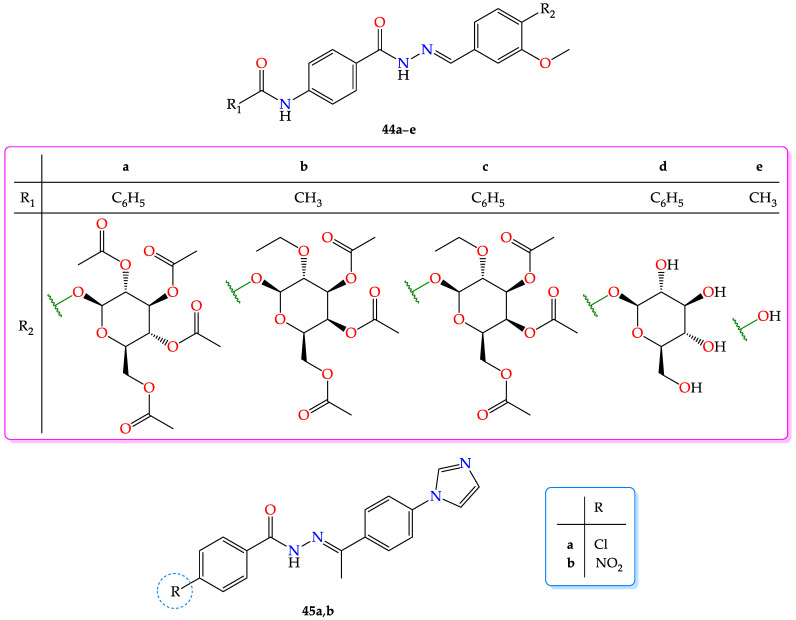
Structures of acylhydrazone derivatives **44a**–**e** and **45a**,**b** with antifungal action.

The acylhydrazones **31a**, **31b**, **31c**, and **31e** (Figure 27) studied on *Candida albicans* exerted a moderate antifungal effect [34]. Additionally, the derivatives **44a**–**e** (Figure 38) showed modest antifungal activity against different fungal strains (*Candida albicans*, *Candida tropicalis*, *Candida krusei*, *Candida glabrata*, and *Candida parapsilosis*) [60]. In the case of compounds **44a**–**d**, the association of the carbohydrate unit with the acylhydrazone moiety determined the increase of the fungicidal effect on *Candida parapsilosis*. The acylhydrazone derivatives **45a**,**b** (Figure 38), from the series synthesized by Reis et al., had selectivity for *Candida glabrata* and a potency comparable to that of the nystatin [61].

**Figure 39:**
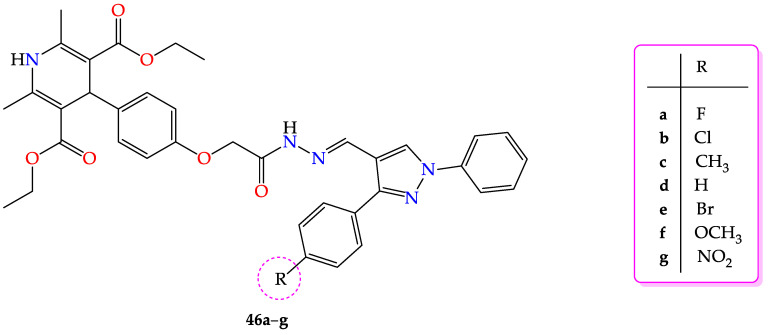
Structures of acylhydrazones **46a**–**g** with antimicrobial and antimalarial actions.

All the compounds **46a**–**g** (Figure 39), obtained by Kumar et al., showed excellent antifungal activity against *Aspergillus niger* compared to the reference drug (clotrimazole), good antimalarial effect against *Plasmodium falciparum* compared to the standard drug chloroquine, and moderate to good antibacterial activity against Gram-positive bacterium strain *Bacillus cereus* compared to clotrimazole [120].

### 5.3. Antiviral Action

**Figure 40:**
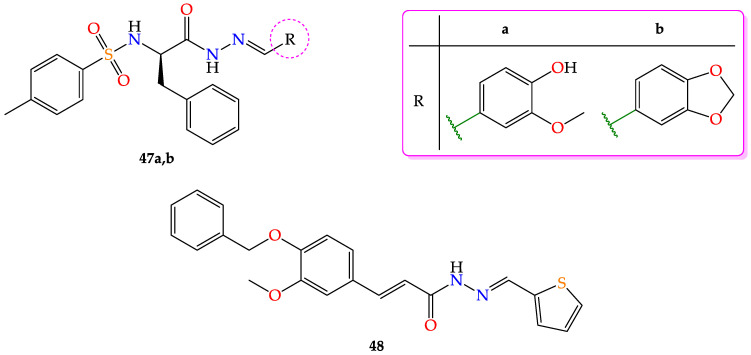
Structures of acylhydrazone derivatives **47a,b** and **48** with antiviral action.

In the case of some derivatives from the acylhydrazone class, it is reported in the literature that they exhibit antiviral action. This effect was identified for acylhydrazones **47a**,**b** and **48** (Figure 40), which were studied as inhibitors targeting *Human immunodeficiency virus type 1* (HIV-1) capsid protein [62] and on *Tobacco mosaic virus*, respectively [63].

**Figure 41:**
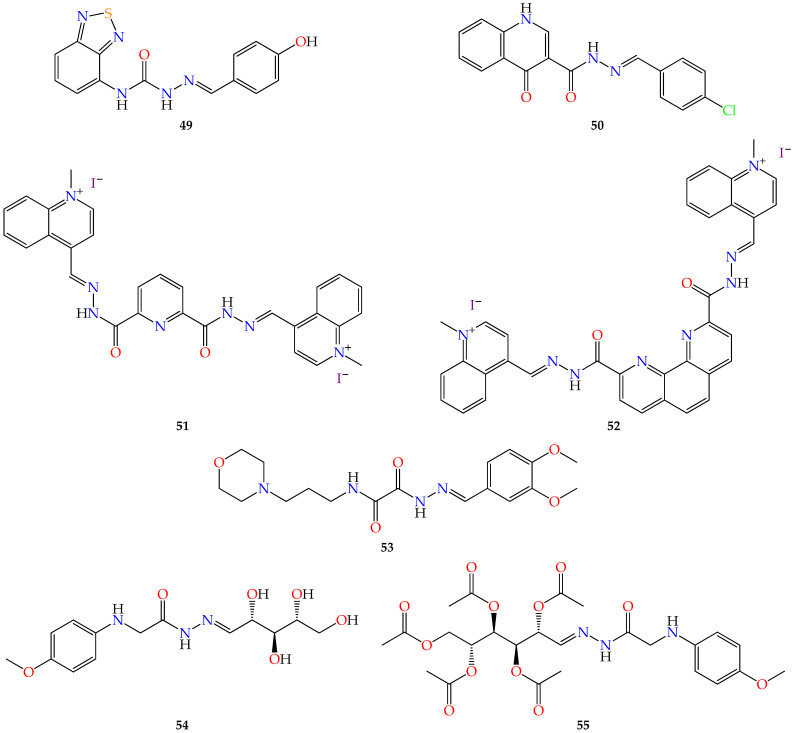
Structures of acylhydrazone derivatives **49**–**55** with antiviral action.

Additionally, the acylhydrazone derivatives **49**–**55** (Figure 41) were studied for their antiviral action. Through the research undertaken, the following results were obtained, namely, compound **49** displayed antiviral action on HIV-1 by blocking the activity of the viral envelope glycoprotein [64], analog **50** showed intense action on HIV-1 [65], and derivatives **51** and **52** had antiviral action on the *Epstein*–*Barr virus* [66]. Compound **53**, with possible application in the treatment of the *Influenza virus*, had neuraminidase inhibitory action more potent than oseltamivir [67]. The derivatives **54** and **55**, containing in their structure a monosaccharide moiety (*D*-mannose, *D*-ribose), displayed the highest potency in the series of studied compounds on *Hepatitis A virus* (**54**) and *Herpes simplex 1* (**55**), using as reference substance amantadine, respectively, acyclovir [68].

In the case of acylhydrazone derivatives, the antiviral action against HIV and *Influenza A virus subtype H1N1* was shown to be determined by the enzymatic inhibition resulting from the chelation of metal ions in the viral structure and endonucleases [69].

**Figure 42:**
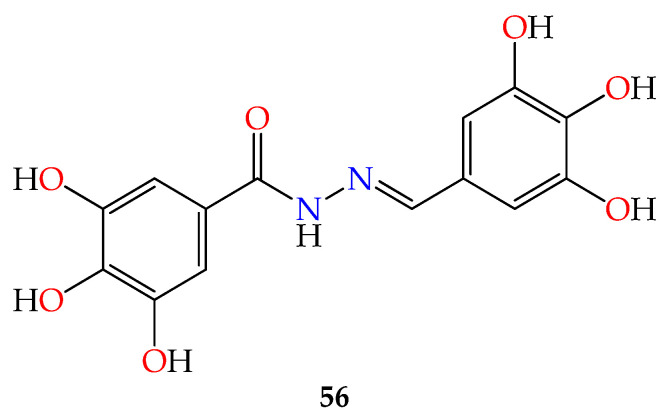
Structure of acylhydrazone **56** with influenza virus endonuclease inhibitory action.

The acylhydrazone class derivative **56** (Figure 42) was found to be an influenza virus endonuclease inhibitor due to the ability of complexation of metal ions (through –OH groups) in the enzyme structure and forming hydrogen bonds [98].

### 5.4. Antiparasitic Action

**Figure 43:**
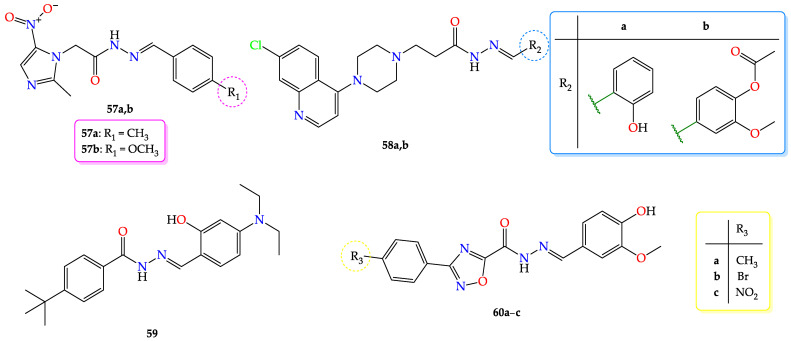
Structures of acylhydrazones **57**–**60** with antiparasitic action.

Some acylhydrazone derivatives were studied for their antiparasitic activity. For example, compounds **57a**,**b** had antiparasitic action against *Entamoeba histolytica* which was superior to that of metronidazole with lower toxicity [6]. Compound **58a** showed antimalarial activity as an inhibitor of β-hematin synthesis and derivative **58b** displayed antiamoebic effect [70]. Compounds **59** [71] and **60a**–**c** exhibited antiparasitic action against the *Plasmodium falciparum*, **60b** being the most potent compound in the series [72]. The structures of acylhydrazone compounds **57**–**60** are presented in Figure 43.

**Figure 44:**
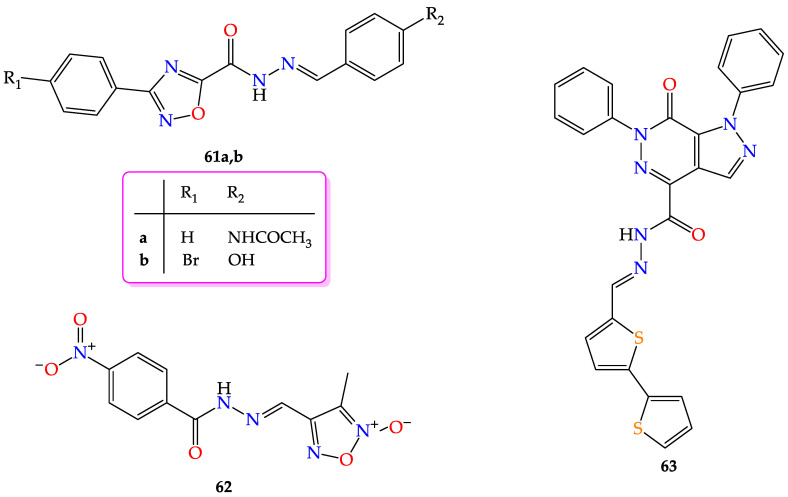
Structures of acylhydrazone derivatives **61**–**63** with antiparasitic action.

The derivatives **61a**,**b** [74], **62** [45] with antiparasitic action on *Trypanosoma cruzi*, and analog **63** [75] active against *Leishmania amazonensis* were also reported (Figure 44).

**Figure 45:**
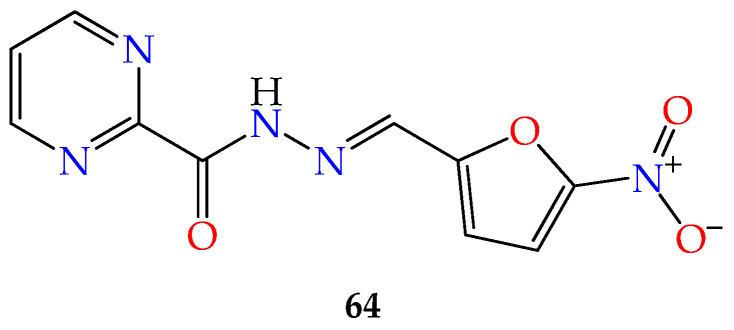
Structure of acylhydrazone **64** with antiparasitic action.

The mechanism of antiparasitic action of the acylhydrazone derivative **64** (Figure 45) is based on membrane depolarization, production of ROS, and alteration of cell membrane integrity in the case of the parasite *L. amazonensis* [121].

**Figure 46:**
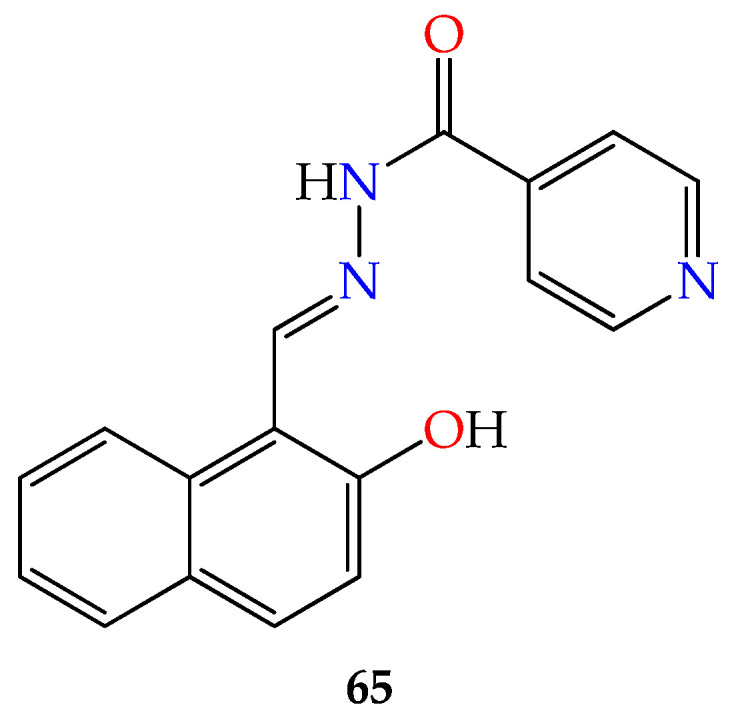
Structure of compound **65** with antiparasitic action on *Plasmodium falciparum*.

A compound with an inhibitory effect on the development of *Plasmodium falciparum* was obtained by complexing the acylhydrazone **65** (Figure 46) with iron ions [73].

**Figure 47:**
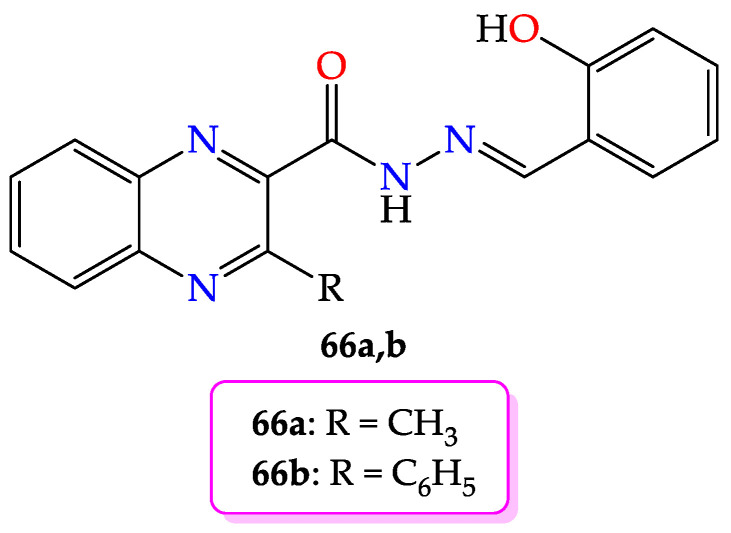
Structures of acylhydrazones **66a**,**b** with antiparasitic action on *Trypanosoma cruzi*.

The acylhydrazones **66a**,**b** (Figure 47) showed antiparasitic action via inhibition of cruzain, the major cysteine protease of *Trypanosoma cruzi*. The effect was comparable to that of the reference substance nifurtimox [76,122].

### 5.5. Anti-Inflammatory Action

**Figure 48:**
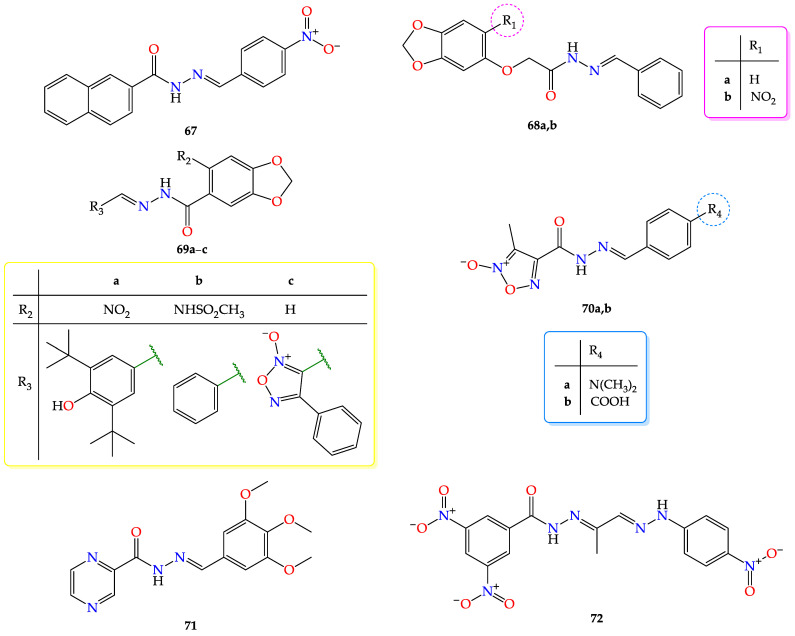
Structures of acylhydrazone derivatives **67**–**72** with anti-inflammatory action.

Acylhydrazone class compounds **67**–**72** (Figure 48) exerted anti-inflammatory activity. Thus, compound **67** inhibited the cascade of arachidonic acid based on the naphthyl group which facilitates hydrophobic interactions with IKK-β [32]. The derivatives **68a**,**b** showed anti-inflammatory and analgesic activities, the effects exerted by **68a** being of lower intensity [77]. In the case of compound **69a**, the anti-inflammatory action was determined by the presence of the –NO_2_ group [78]. The derivative **69b** had an anti-inflammatory action comparable to that of nimesulide [79]. Compounds **69c** and **70a**,**b** demonstrated the anti-inflammatory effect by inhibiting the NF-kB pathway and the release of IL-8 [80]. Analog **71** also exerted analgesic action in addition to the anti-inflammatory one [81]. The derivative **72** had an anti-inflammatory effect by reducing the eosinophilia due to low IL-4, IL-5, and IL-13 cytokine levels [82]. This suggests its therapeutic potential for treating allergic diseases. Additionally, **72** demonstrated the anti-inflammatory action by modulating IL-1β secretion and PGE2 synthesis in macrophages and by inhibiting calcineurin phosphatase activity in lymphocytes [83].

**Figure 49:**
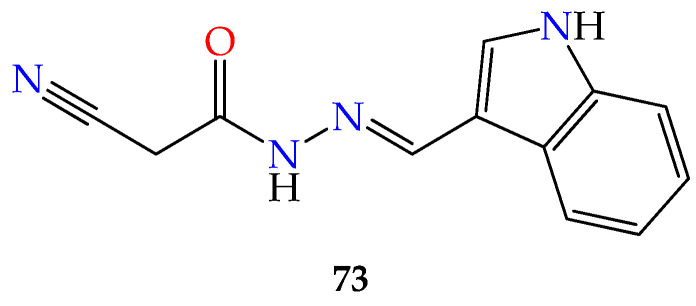
Structure of the acylhydrazone derivative **73** with anti-inflammatory action.

The anti-inflammatory effect of acylhydrazone **73** (Figure 49) was due to the selective inhibition of cyclooxygenase-2 (COX-2) and decreasing lymphocyte proliferation [84]. Moreover, the in silico analysis and experimental results suggested that **73** exhibits a well-balanced pharmacodynamic and pharmacokinetic profile.

**Figure 50:**
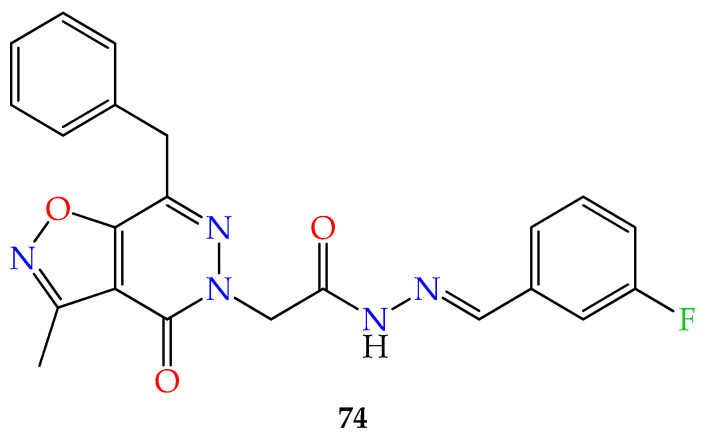
Structure of the acylhydrazone derivative **74** with non-selective COX inhibitory action.

Compound **74** (Figure 50), synthesized by Ünsal-Tan et al., was reported as a non-selective COX inhibitor with the highest potency among the studied derivatives [97].

**Figure 51:**
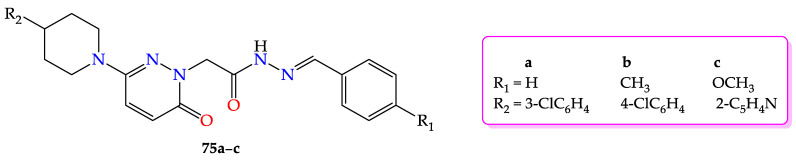
Structures of compounds **75a**–**c** with anti-inflammatory action.

The acylhydrazones **75a**–**c** (Figure 51) exhibited an anti-inflammatory effect comparable to that of indomethacin, but do not affect the gastric mucosa [73].

In addition to the anti-inflammatory activity, compounds of the acylhydrazone class **68a**,**b** [77], **69a** [78], **69b** [79], **70a** [80], and **71** [81] (Figure 48) demonstrated analgesic action. This effect, in association with the anti-inflammatory activity, may have possible therapeutic applications in various pathologies.

**Figure 52:**
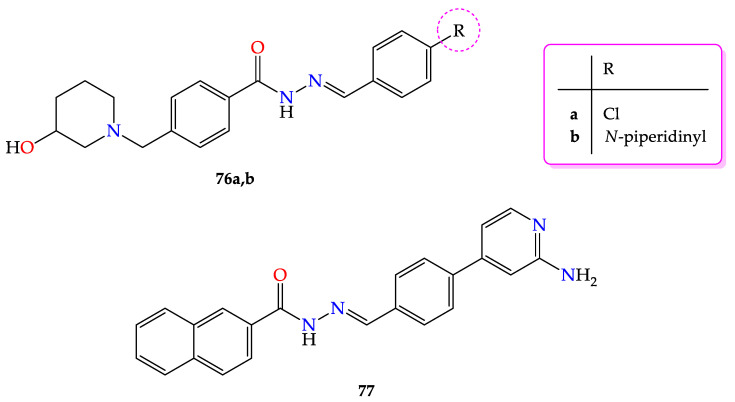
Structures of acylhydrazones **76a**,**b** and **77** with anti-inflammatory and analgesic actions.

It was found that the analgesic action mediated by acylhydrazones **76a**,**b** was exerted via the opioidergic system [36]. Cordeiro et al. showed that amino-pyridinyl-*N*-acylhydrazone **77** exhibited anti-inflammatory activity by inhibiting p38α, reducing inflammatory pain, cell migration, and inflammatory mediators participating in the MAPK pathway, such as IL-1β and NF-α [123]. The structures of acylhydrazones **76a**,**b** and **77** are presented in Figure 52.

### 5.6. Immunomodulatory Action

The action of acylhydrazone derivatives on the immune system was also reported in the literature. The acylhydrazone class derivative **72** (Figure 48) showed immunomodulatory effect by inhibiting cytokine production and lymphocyte proliferation [83].

**Figure 53:**
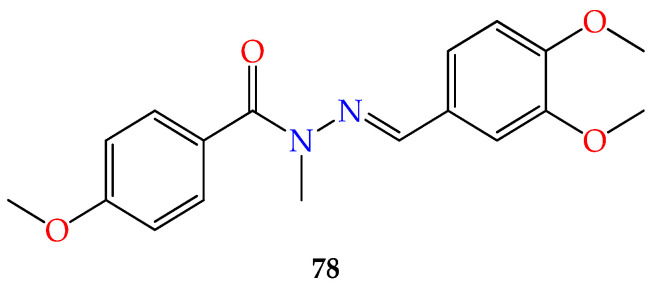
Structure of acylhydrazone **78** with immunosuppressive effect.

According to a study conducted by Guimarães et al., acylhydrazone **78** (Figure 53) exhibited immunosuppressive activity due to the inhibitory action of phosphodiesterase-4 (PDE-4), inhibiting phosphorylation of IkB protein which interferes with the NF-kB pathway [86].

### 5.7. Antiedematous Action

Compounds **25b** (R_1_ = C_6_H_5_, R_2_ = 2-pyridyl) and **25c** (R_1_ = CH_3_, R_2_ = 4-BrC_6_H_4_) (Figure 21), having in their structures the acylhydrazone moiety closed in a heterocycle, showed antiedematous effect by inhibiting carbonic anhydrase I isoform [29].

### 5.8. Antiglaucomatous Action

**Figure 54:**
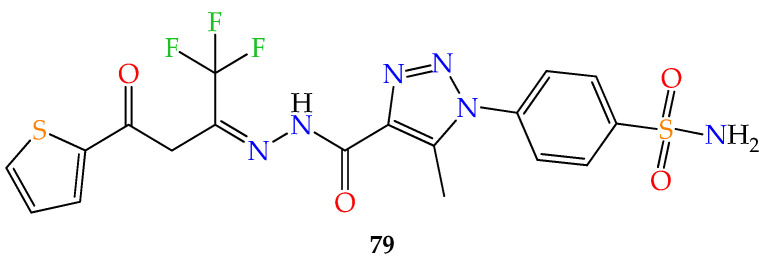
Structure of acylhydrazone compound **79** with antiglaucomatous effect.

The acylhydrazone derivatives **25c** (R_1_ = CH_3_ and R_2_ = 4-BrC_6_H_4_), **25d** (R_1_ = CH_3_ and R_2_ = C_6_H_5_), **25e** (R_1_ = CH_3_ and R_2_ = 4-CH_3_C_6_H_4_) (Figure 21), and **79** (Figure 54) exhibited antiglaucomatous activity by inhibiting the carbonic anhydrase II isoform [29].

### 5.9. Activity on the Central Nervous System (CNS)

**Figure 55:**
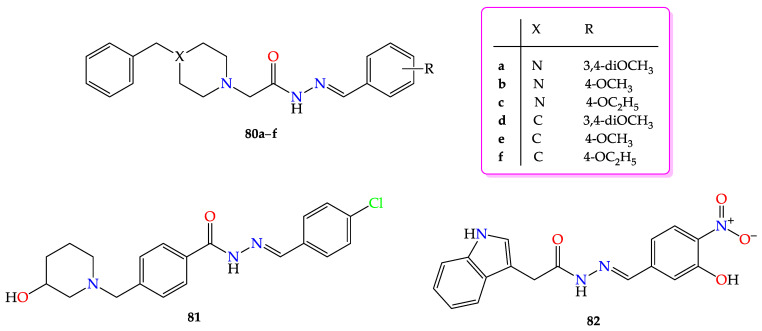
Structures of compounds **80**–**82** with activity on the CNS.

The acylhydrazones **80a**–**f** showed the inhibitory action on acetylcholinesterase and good antiaggregation activity on plates of β-amyloid. The enzyme inhibition was noted as the effect depending on the conformation of the enzyme-substrate complex, with relatively better results than the other compounds in the case of **80d** and **80f** [88]. The derivative **81** is one of the substances synthesized by Viegas et al. with possible beneficial effects in Alzheimer’s disease by inhibiting acetylcholinesterase, COX-1, and COX-2 [89]. Compound **82** could be a potential candidate for use in neurodegenerative diseases due to passive diffusion through the blood–brain barrier and controlling neuronal synapses [90]. The structures of compounds **80**–**82** are presented in Figure 55.

**Figure 56:**
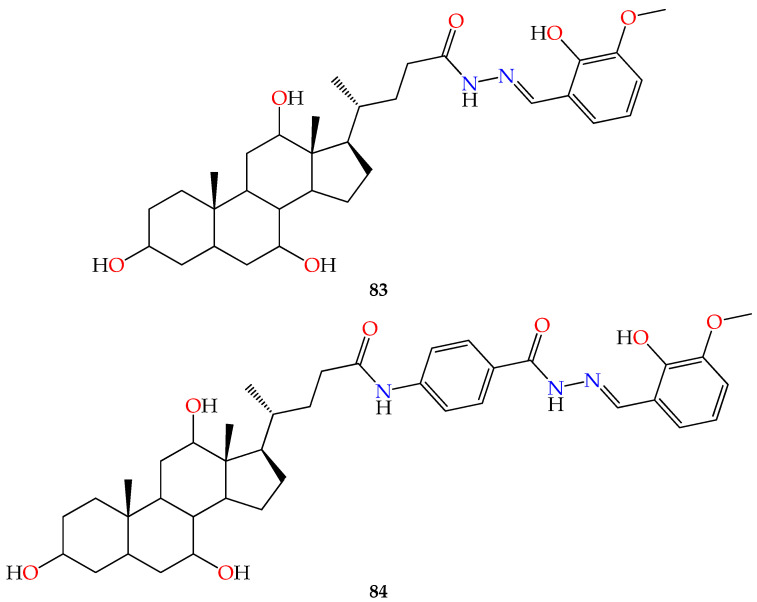
Structures of acylhydrazones **83** and **84** with action due to chelation of iron (II) ion.

Compounds **83** and **84** (Figure 56), which showed the chelating affinity of iron (II) ions, presented inhibitory action on ten-eleven translocation methylcytosine dioxygenase 1 (TET 1). This protein catalyzes the chemical reaction of transforming 5-methylcytosine into 5-hydroxymethylcytosine, a substance that in abnormal concentrations is associated with diverse pathologies, like leukemia, Parkinson’s disease, and Alzheimer’s disease [87].

**Figure 57:**
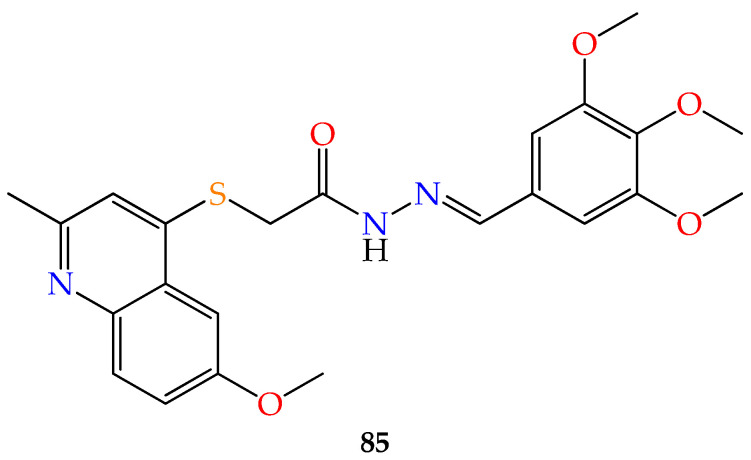
Structure of acylhydrazone **85** with PDE10A inhibitory action.

An acylhydrazone derivative **85** (Figure 57) was reported as a potent phosphodiesterase 10A (PDE10A) inhibitor probably due to the presence in their structure of the substituted 4-quinoline nucleus [92]. The PDE10A is implicated in diverse central nervous system pathologies, such as Parkinson’s disease and Huntington’s disease, and mental disorders, like schizophrenia [92].

**Figure 58:**
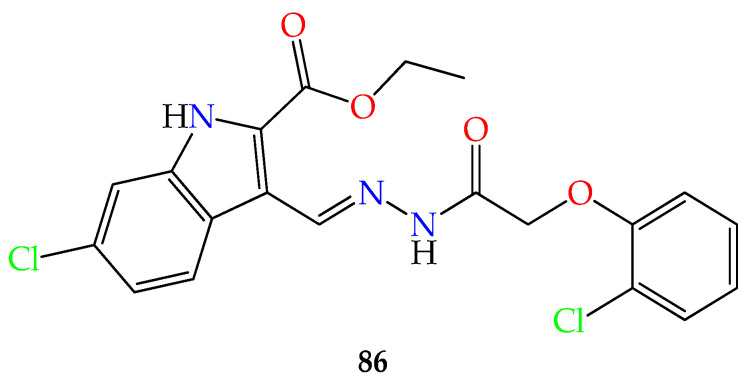
Structure of acylhydrazone **86** with inhibitory action of a LOX isoform.

The derivative of acylhydrazone **86** (Figure 58) exerted inhibitory action of an isoform of lipooxygenase (15-LOX-1) with good intensity, due to the *ortho*-chlorine atom on the benzene nucleus. This isoform is involved in pathologies, such as Alzheimer’s disease and Parkinson’s disease [95].

**Figure 59:**
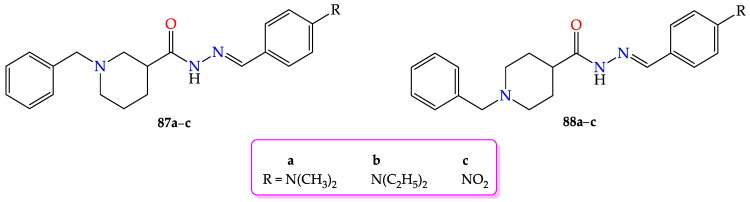
Structures of acylhydrazone derivatives **87a**–**c** and **88a**–**c** with antioxidant action.

The piperidinehydrazide-hydrazones **87a**–**c** and **88a**–**c** (Figure 59) showed potential anti-Alzheimer activity by inhibiting the β-amyloid plaque formation [99]. Furthermore, the acylhydrazones **87a**,**b** and **88a**,**b** displayed strong antioxidant activity due to the presence in their molecules of the dimethylamino (**87a**, **88a**), respectively, diethylamino moiety (**87b**, **88b**).

### 5.10. Antidiabetic Activity

**Figure 60:**
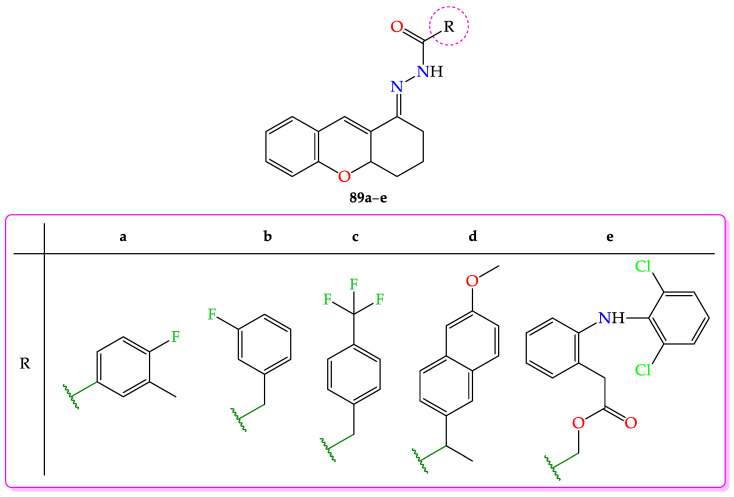
Structures of acylhydrazone compounds **89a**–**e** with antidiabetic action.

Compounds **89a**–**e** (Figure 60) showed an antidiabetic effect due to the inhibition of α-glucosidase, an enzyme that catalyzes the cleavage of oligosaccharides into monosaccharides. The derivative with an electronegative group in the *para* position (**89c**) exhibited the most intense action [18].

### 5.11. Antioxidant Action

Among the various pharmacological studies performed in the case of some acylhydrazone derivatives are those that showed their antioxidant effects [34,39,78,101].

In addition to the above-mentioned properties identified in the case of acylhydrazones **31a**, **31c**, **31g** (Figure 27) [34], and **69a** (Figure 48) [78], the antioxidant action of the specified derivatives was also reported. This activity was tested using DPPH [34,78], ferric-reducing antioxidant power (FRAP) [34], hydroxyl-mediated deoxyribose degradation, and superoxide radical scavenging assays [78].

**Figure 61:**
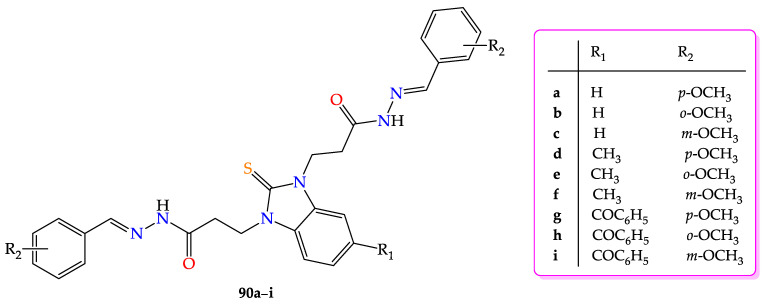
Structures of acylhydrazone class derivatives **90a**–**i** with antioxidant action.

The antioxidant action was reported for the compounds **90a**–**i** (Figure 61) using the oxidative stress induced by *tert*-butyl hydroperoxide. Moreover, the cytoprotective effect was investigated, which indicated that derivatives **90a**–**c** and **90g**–**i** showed effects comparable to those of the reference substance (quercetin), and compounds **90d**–**f** exhibited weaker effects compared to this one [100].

### 5.12. Action on the Cardiovascular System

**Figure 62:**
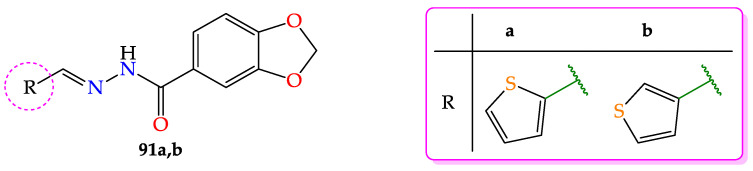
Structures of acylhydrazones **91a**,**b** with action on the cardiovascular system.

Among the different biological properties and possible therapeutic indications of the acylhydrazone class compounds, their actions on the cardiovascular system were identified. Thus, acylhydrazone **91a** (Figure 62) may be a potential candidate for use in the treatment scheme of cardiac remodeling, respectively in combating diastolic disorders after myocardial infarction. This derivative has the potential to reduce cardiac remodeling after myocardial infarction by regulating inflammatory mediators, leading to reduced inflammation and cardiac fibrosis. The positive inotropic effect of compound **91a** was observed by stimulating the activity of the sarcoplasmic/endoplasmic reticulum Ca^2+^-ATPase 2a (SERCA2a) protein, causing both the uptake of Ca^2+^ ions into the sarcoplasmic reticulum and intracellular Ca^2+^ utilization. This effect was also observed in healthy cardiomyocytes by increasing the intracellular Ca^2+^ concentration. Compound **91a** regulates the phosphorylation and dephosphorylation of troponin I, troponin T, and protein C, respectively, but the Ca^2+^ sensitivity of contractile proteins was not noted in this study. Thus, this analog is considered a promising agent for use in the treatment of heart failure after myocardial infarction [102]. The above-mentioned acylhydrazone was also found to prevent exercise intolerance after myocardial infarction, probably by producing NO with vasodilating action by increasing the level of cyclic guanosine monophosphate (cGMP) in vascular smooth muscle cells and by activating adenosine A_2A_ receptors leading to the decreased inflammatory response. Compound **91a** could thus increase the blood flow to muscles, prevent the oxidation of proteins, and reduce the pro-inflammatory cytokines, which could lead to improved skeletal muscle contractile response after myocardial infarction [103].

The acylhydrazone derivative **91b** had a vasodilating effect, by increasing the concentrations of NO and cGMP, more potent than its isomer with possible use in the treatment scheme of hypertension. Additionally, compound **91b** is an M_3_ muscarinic receptor agonist proved by the antagonist effect of a selective antagonist, 4-diphenylacetoxy-*N*-methylpiperidine methiodide. The acylhydrazone **91b** had a reduced number of adverse reactions compared to other parasympathomimetic compounds. This derivative was reported for its hypotensive effect with no modification in heart rate observed for both intravenous and longer-term oral administration with possible use in the treatment of hypertension [104]. The structure of compound **91b** [124] is shown in Figure 62.

**Figure 63:**
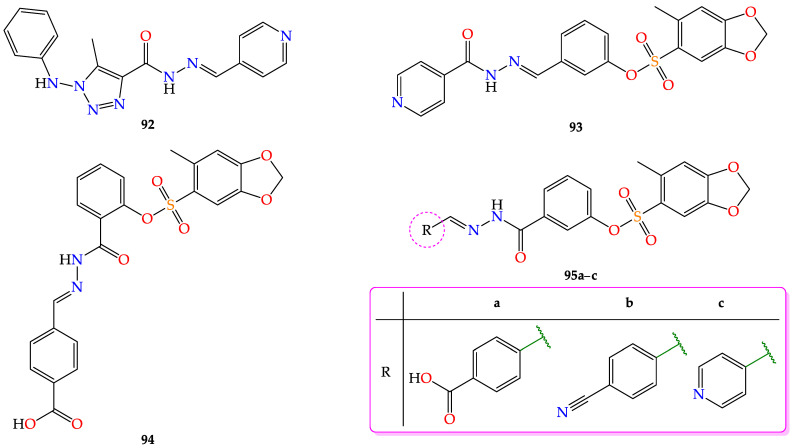
Structures of derivatives **92**–**95** showing antiplatelet action.

Sathler et al. obtained an acylhydrazone **92** that demonstrated antithrombotic properties when collagen was used as an agonist and lower toxicity compared to other derivatives. The proposed mechanism is based on the interaction of the compound with TXA_2_ synthase, acting as an inhibitor [105]. Additionally, Lima et al. synthesized the arylsulfonate–acylhydrazone derivatives **93**–**95** with antiplatelet activity [106]. The structures of the acylhydrazones **92**–**95** are presented in Figure 63.

**Figure 64:**
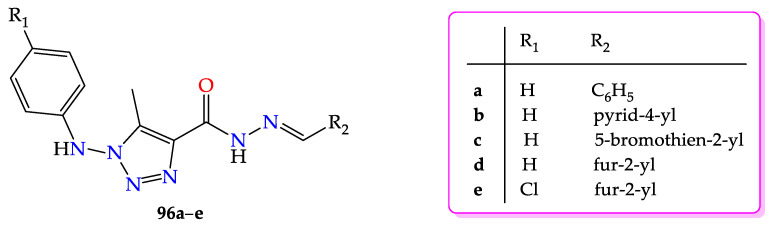
Structures of compounds **96a**–**e** with antithrombotic action.

Other derivates with antiplatelet effect are acylhydrazones containing the 1,2,3-triazole scaffold **96a**–**e** (Figure 64), which exhibited a comparable or even higher potency than acetylsalicylic acid. The inhibitory activity observed in the arachidonic acid test was different in the case of studied compounds due to the various structural fragments, as follows: **96a**—adenosine diphosphate (ADP) pathway antagonist, **96a**,**c**,**d**,**e**—adrenaline pathway antagonists, and **96b**,**c**,**e**—arachidonic acid pathway antagonists [107].

**Figure 65:**
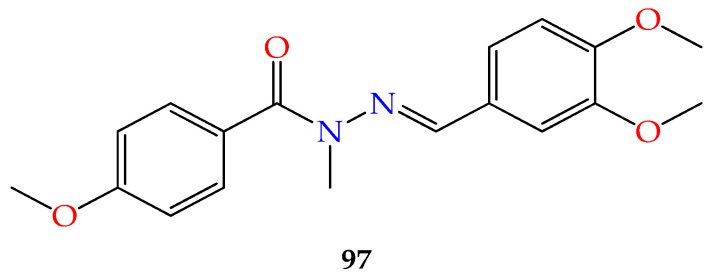
Structure of acylhydrazone **97** with action on ventricular remodeling.

According to a research study conducted by Alencar et al., an acylhydrazone derivative **97** (Figure 65) was analyzed pharmacologically. It lowered the pressure on the pulmonary arteries by interacting with adenosine A_2A_ receptors, which have an important role in the pathophysiological mechanism of pulmonary arterial hypertension. Thus, the acylhydrazone **97** had an effect on ventricular remodeling (right ventricular hypertrophy) by decreasing it, lowering the right ventricular systolic pressure, stimulating SERCA2a protein and endothelial nitric oxide synthase, reducing the levels of phospholamban [108].

**Figure 66:**
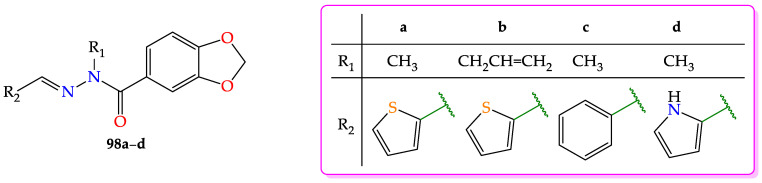
Structures of acylhydrazone class derivatives **98a**–**d** with vasodilatory action.

Silva et al. stated that the derivatives of acylhydrazones **98a** and **98b** (Figure 66) displayed vasodilatory action. Compound **98b**, containing an allyl moiety linked to the amide nitrogen atom, showed a potency equivalent to that of compound **91a** (Figure 62) and of acylhydrazone **98a**, with a methyl group substituting the amide hydrogen atom [109]. The same biological property was exerted by compounds **98c** and **98d** (Figure 66), the vasodilatory action being more intense than that of acylhydrazone **91a** [110].

**Figure 67:**
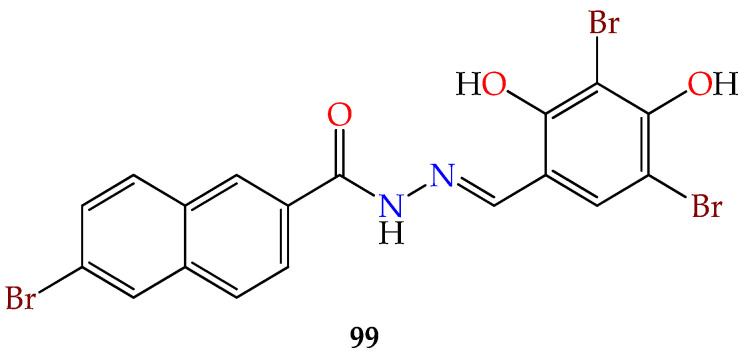
Structure of compound **99** with possible use in ischemic stroke.

The acylhydrazone **99** (Figure 67) was reported by Feng et al. as a substance that can protect cells from oxygen-glucose deprivation, oxidative stress stimulated by H_2_O_2_ and glutamate, stimulated apoptosis by oxygen-glucose deprivation, increased intracellular ROS, and increased ATP levels in neuronal cells. Compound **99** also increased the phosphorylation based on extracellular signal-regulated kinase and protein kinase B, based on antagonistic action with selective antagonists, and had favorable effects on stroke, inducing neuroprotection. Therefore, acylhydrazone **99** could be used in ischemic strokes after further research [111].

## References

[1] Gao P., Wei Y. (2013). Efficient oxidative cyclization of N-acylhydrazones for the synthesis of 2,5-disubstituted 1,3,4-oxadiazoles using t-BuOI under neutral conditions. Heterocycl. Commun..

[2] Başpınar Küçük H., Alhonaish A., Yıldız T., Güzel M. (2022). An efficient approach to access 2,5-disubstituted 1,3,4-oxadiazoles by oxidation of 2-arenoxybenzaldehyde N- acyl hydrazones with molecular iodine. ChemistrySelect.

[3] Pelipko V.V., Gomonov K.A. (2021). Formation of five- and six-membered nitrogen-containing heterocycles on the basis of hydrazones derived from α-dicarbonyl compounds (microreview). Chem. Heterocycl. Compd..

[4] Morjan R.Y., Mkadmh A.M., Beadham I., Elmanama A.A., Mattar M.R., Raftery J., Pritchard R.G., Awadallah A.M., Gardiner J.M. (2014). Antibacterial activities of novel nicotinic acid hydrazides and their conversion into N-acetyl-1,3,4-oxadiazoles. Bioorganic Med. Chem. Lett..

[5] Ţînţaş M.L., Diac A.P., Soran A., Terec A., Grosu I., Bogdan E. (2014). Structural characterization of new 2-aryl-5-phenyl-1,3,4-oxadiazin-6-ones and their N-aroylhydrazone precursors. J. Mol. Struct..

[6] Wani M.Y., Bhat A.R., Azam A., Athar F. (2013). Nitroimidazolyl hydrazones are better amoebicides than their cyclized 1,3,4-oxadiazoline analogues: In vitro studies and Lipophilic efficiency analysis. Eur. J. Med. Chem..

[7] Mali S.N., Thorat B.R., Gupta D.R., Pandey A. (2021). Mini-Review of the Importance of Hydrazides and Their Derivatives—Synthesis and Biological Activity. Eng. Proc..

[8] de Oliveira Carneiro Brum J., França T.C.C., LaPlante S.R., Villar J.D.F. (2020). Synthesis and Biological Activity of Hydrazones and Derivatives: A Review. Mini-Rev. Med. Chem..

[9] Thota S., Rodrigues D.A., de Sena Murteira Pinheiro P., Lima L.M., Fraga C.A.M., Barreiro E.J. (2018). N-Acylhydrazones as drugs. Bioorganic Med. Chem. Lett..

[10] Demir Y., Tokalı F.S., Kalay E., Türkeş C., Tokalı P., Aslan O.N., Şendil K., Beydemir Ş. (2022). Synthesis and characterization of novel acyl hydrazones derived from vanillin as potential aldose reductase inhibitors. Mol. Divers..

[11] Cui P., Cai M., Meng Y., Yang Y., Song H., Liu Y., Wang Q. (2022). Design, synthesis and biological activities of echinopsine derivatives containing acylhydrazone moiety. Sci. Rep..

[12] Zhou Y., Zhang S., He H., Jiang W., Hou L., Xie D., Cai M., Peng H., Feng L. (2018). Design and synthesis of highly selective pyruvate dehydrogenase complex E1 inhibitors as bactericides. Bioorganic Med. Chem..

[13] Lawrence M.A.W., Lorraine S.C., Wilson K.-A., Wilson K. (2019). Review: Voltammetric properties and applications of hydrazones and azo moieties. Polyhedron.

[14] Purandara H., Raghavendra S., Foro S., Patil P., Gowda B.T., Dharmaprakash S.M., Vishwanatha P. (2019). Synthesis, spectroscopic characterization, crystal structure, Hirshfeld surface analysis and third-order nonlinear optical properties of 2-(4-chlorophenoxy)-N’-[(1E)-1-(4-methylphenyl) ethylidene] acetohydrazide. J. Mol. Struct..

[15] Liu Y., Di Y., Di Y., Qiao C., Chen F., Yuan F., Yue K., Zhou C. (2019). The studies of structure, thermodynamic properties and theoretical analyses of 2-[(4-nitro-benzoyl)-hydrazone]-propionic acid. J. Mol. Struct..

[16] Selvam P., Sathiyakumar S., Srinivasan K., Premkumar T. (2019). A Copper(II) complex of a new hydrazone: A solid-state single source precursor for the preparation of both Cu and CuO nanoparticles. J. Mol. Struct..

[17] Sadhukhan D., Maiti M., Bauzá A., Frontera A., Garribba E., Gomez-García C.J. (2019). Synthesis, structure, physicochemical characterization and theoretical evaluation of non-covalent interaction energy of a polymeric copper(II)-hydrazone complex. Inorg. Chim. Acta.

[18] Tariq Q.-N., Malik S., Khan A., Naseer M.M., Khan S.U., Ashraf A., Ashraf M., Rafiq M., Mahmood K., Tahir M.N. (2019). Xanthenone-based hydrazones as potent α-glucosidase inhibitors: Synthesis, solid state self-assembly and in silico studies. Bioorganic Chem..

[19] Kuriakose D., Kurup M.R.P. (2019). Crystal structures and supramolecular architectures of ONO donor hydrazone and solvent exchangeable dioxidomolybdenum(VI) complexes derived from 3,5-diiodosalicyaldehyde-4-methoxybenzoylhydrazone: Hirshfeld surface analysis and interaction energy calculat. Polyhedron.

[20] Yang H.-L., Dang Z.-J., Zhang Y.-M., Wei T.-B., Yao H., Zhu W., Fan Y.-Q., Jiang X.-M., Lin Q. (2019). Novel cyanide supramolecular fluorescent chemosensor constructed from a quinoline hydrazone functionalized-pillar[5]arene. Spectrochim. Acta Part A Mol. Biomol. Spectrosc..

[21] Gamov G.A., Khodov I.A., Belov K.V., Zavalishin M.N., Kiselev A.N., Usacheva T.R., Sharnin V.A. (2019). Spatial structure, thermodynamics and kinetics of formation of hydrazones derived from pyridoxal 5′-phosphate and 2-furoic, thiophene-2-carboxylic hydrazides in solution. J. Mol. Liq..

[22] Hincapié-Otero M.M., Joaqui-Joaqui A., Polo-Cerón D. (2021). Synthesis and characterization of four N-acylhydrazones as potential O,N,O donors for Cu2+: An experimental and theoretical study. Univ. Sci..

[23] Munir R., Javid N., Zia-ur-Rehman M., Zaheer M., Huma R., Roohi A., Athar M.M. (2021). Synthesis of Novel N-Acylhydrazones and Their C-N/N-N Bond Conformational Characterization by NMR Spectroscopy. Molecules.

[24] García-Ramírez V.G., Suarez-Castro A., Villa-Lopez M.G., Díaz-Cervantes E., Chacón-García L., Cortes-García C.J. (2020). Synthesis of Novel Acylhydrazone-Oxazole Hybrids and Docking Studies of SARS-CoV-2 Main Protease. Chem. Proc..

[25] Aneja B., Khan N.S., Khan P., Queen A., Hussain A., Rehman M.T., Alajmi M.F., El-Seedi H.R., Ali S., Hassan M.I. (2019). Design and development of Isatin-triazole hydrazones as potential inhibitors of microtubule affinity-regulating kinase 4 for the therapeutic management of cell proliferation and metastasis. Eur. J. Med. Chem..

[26] Salum L.B., Mascarello A., Canevarolo R.R., Altei W.F., Laranjeira A.B.A., Neuenfeldt P.D., Stumpf T.R., Chiaradia-Delatorre L.D., Vollmer L.L., Daghestani H.N. (2015). N-(1’-naphthyl)-3,4,5-trimethoxybenzohydrazide as microtubule destabilizer: Synthesis, cytotoxicity, inhibition of cell migration and in vivo activity against acute lymphoblastic leukemia. Eur. J. Med. Chem..

[27] Govindaiah P., Dumala N., Mattan I., Grover P., Jaya Prakash M. (2019). Design, synthesis, biological and in silico evaluation of coumarin-hydrazone derivatives as tubulin targeted antiproliferative agents. Bioorganic Chem..

[28] Sreenivasulu R., Reddy K.T., Sujitha P., Kumar C.G., Raju R.R. (2019). Synthesis, antiproliferative and apoptosis induction potential activities of novel bis(indolyl)hydrazide-hydrazone derivatives. Bioorganic Med. Chem..

[29] Sharma V., Kumar R., Bua S., Supuran C.T., Sharma P.K. (2019). Synthesis of novel benzenesulfonamide bearing 1,2,3-triazole linked hydroxy-trifluoromethylpyrazolines and hydrazones as selective carbonic anhydrase isoforms IX and XII inhibitors. Bioorganic Chem..

[30] Chivers P.R.A., Smith D.K. (2019). Shaping and structuring supramolecular gels. Nat. Rev. Mater..

[31] Rezki N., Al-Sodies S.A., Ahmed H.E.A., Ihmaid S., Messali M., Ahmed S., Aouad M.R. (2019). A novel dicationic ionic liquids encompassing pyridinium hydrazone-phenoxy conjugates as antimicrobial agents targeting diverse high resistant microbial strains. J. Mol. Liq..

[32] Avila C.M., Lopes A.B., Gonçalves A.S., da Silva L.L., Romeiro N.C., Miranda A.L.P., Sant’Anna C.M.R., Barreiro E.J., Fraga C.A.M. (2011). Structure-based design and biological profile of (E)-N-(4-Nitrobenzylidene)-2-naphthohydrazide, a novel small molecule inhibitor of IκB kinase-β. Eur. J. Med. Chem..

[33] Rohane S.H., Chauhan A.J., Fuloria N.K., Fuloria S. (2020). Synthesis and in vitro antimycobacterial potential of novel hydrazones of eugenol. Arab. J. Chem..

[34] Siddique M., Bin Saeed A., Dogar N.A., Ahmad S. (2013). Biological Potential of Synthetic Hydrazide Based Schiff Bases. J. Sci. Innov. Res..

[35] Xia L., Xia Y.-F., Huang L.-R., Xiao X., Lou H.-Y., Liu T.-J., Pan W.-D., Luo H. (2015). Benzaldehyde Schiff bases regulation to the metabolism, hemolysis, and virulence genes expression in vitro and their structure–microbicidal activity relationship. Eur. J. Med. Chem..

[36] dos Santos N.M., Pereira N.C., de Albuquerque A.P.S., Dias Viegas F.P., Veloso C., Vilela F.C., Giusti-Paiva A., da Silva M.L., da Silva J.R.T., Viegas C. (2018). 3-Hydroxy-piperidinyl-N-benzyl-acyl-arylhydrazone derivatives reduce neuropathic pain and increase thermal threshold mediated by opioid system. Biomed. Pharmacother..

[37] Chen D.-M., Wu X.-F., Liu Y.-J., Huang C., Zhu B.-X. (2019). Synthesis, crystal structures and vapor adsorption properties of Hg(II) and Cd(II) coordination polymers derived from two hydrazone Schiff base ligands. Inorg. Chim. Acta.

[38] Singh A.K., Thakur S., Pani B., Chugh B., Lgaz H., Chung I.-M., Chaubey P., Pandey A.K., Singh J. (2019). Solvent-free microwave assisted synthesis and corrosion inhibition study of a series of hydrazones derived from thiophene derivatives: Experimental, surface and theoretical study. J. Mol. Liq..

[39] Demurtas M., Baldisserotto A., Lampronti I., Moi D., Balboni G., Pacifico S., Vertuani S., Manfredini S., Onnis V. (2019). Indole derivatives as multifunctional drugs: Synthesis and evaluation of antioxidant, photoprotective and antiproliferative activity of indole hydrazones. Bioorganic Chem..

[40] Lis C., Rubner S., Roatsch M., Berg A., Gilcrest T., Fu D., Nguyen E., Schmidt A.-M., Krautscheid H., Meiler J. (2017). Development of Erasin: A chromone-based STAT3 inhibitor which induces apoptosis in Erlotinib-resistant lung cancer cells. Sci. Rep..

[41] Sun K., Peng J.-D., Suo F.-Z., Zhang T., Fu Y.-D., Zheng Y.-C., Liu H.-M. (2017). Discovery of tranylcypromine analogs with an acylhydrazone substituent as LSD1 inactivators: Design, synthesis and their biological evaluation. Bioorganic Med. Chem. Lett..

[42] Congiu C., Onnis V. (2013). Synthesis and biological evaluation of novel acylhydrazone derivatives as potential antitumor agents. Bioorganic Med. Chem..

[43] Cui Z., Li Y., Ling Y., Huang J., Cui J., Wang R., Yang X. (2010). New class of potent antitumor acylhydrazone derivatives containing furan. Eur. J. Med. Chem..

[44] Li Y., Yan W., Yang J., Yang Z., Hu M., Bai P., Tang M., Chen L. (2018). Discovery of novel β-carboline/acylhydrazone hybrids as potent antitumor agents and overcome drug resistance. Eur. J. Med. Chem..

[45] Massarico Serafim R.A., Gonçalves J.E., de Souza F.P., de Melo Loureiro A.P., Storpirtis S., Krogh R., Andricopulo A.D., Dias L.C., Ferreira E.I. (2014). Design, synthesis and biological evaluation of hybrid bioisoster derivatives of N-acylhydrazone and furoxan groups with potential and selective anti-Trypanosoma cruzi activity. Eur. J. Med. Chem..

[46] Yao Q., Qi J., Zheng Y., Qian K., Wei L., Maimaitiyiming M., Cheng Z., Wang Y. (2019). Synthesis, anticancer activity and mechanism of iron chelator derived from 2,6-diacetylpyridine bis(acylhydrazones). J. Inorg. Biochem..

[47] Yu X., Shi L., Ke S. (2015). Acylhydrazone derivatives as potential anticancer agents: Synthesis, bio-evaluation and mechanism of action. Bioorganic Med. Chem. Lett..

[48] Duan Y.-T., Man R.-J., Tang D.-J., Yao Y.-F., Tao X.-X., Yu C., Liang X.-Y., Makawana J.A., Zou M.-J., Wang Z.-C. (2016). Design, Synthesis and Antitumor Activity of Novel link-bridge and B-Ring Modified Combretastatin A-4 (CA-4) Analogues as Potent Antitubulin Agents. Sci. Rep..

[49] Barbosa V.A., Formagio A.S.N., Savariz F.C., Foglio M.A., Spindola H.M., de Carvalho J.E., Meyer E., Sarragiotto M.H. (2011). Synthesis and antitumor activity of β-carboline 3-(substituted-carbohydrazide) derivatives. Bioorganic Med. Chem..

[50] de Almeida P.S.V.B., Pereira T.M., Kummerle A.E., Guedes G.P., Silva H., de Oliveira L.L., Neves A.P. (2019). New Ru(II)–DMSO complexes containing coumarin-N-acylhydrazone hybrids: Synthesis, X-ray structures, cytotoxicity and antimicrobial activities. Polyhedron.

[51] Liu X., Tang Y., He X., Ge X., Liu J., Meng X., Shao M., Jin Y., Tian L., Liu Z. (2019). Triphenyltin(IV) acylhydrazone compounds: Synthesis, structure and bioactivity. J. Inorg. Biochem..

[52] Socea L.-I., Socea B., Saramet G., Barbuceanu S.-F., Draghici C., Constantin V.D., Olaru O.T. (2015). Synthesis and Cytotoxicity Evaluation of new 5H-dibenzo[a,d][7]annulen-5-yl acetylhydrazones. Rev. Chim..

[53] He H., Xia H., Xia Q., Ren Y., He H. (2017). Design and optimization of N-acylhydrazone pyrimidine derivatives as E. coli PDHc E1 inhibitors: Structure-activity relationship analysis, biological evaluation and molecular docking study. Bioorganic Med. Chem..

[54] Jin Y.-X., Zhong A.-G., Ge C.-H., Pan F.-Y., Yang J.-G., Wu Y., Xie M., Feng H.-W. (2012). A novel difunctional acylhydrazone with isoxazole and furan heterocycles: Syntheses, structure, spectroscopic properties, antibacterial activities and theoretical studies of (E)-N′-(furan-2-ylmethylene)-5-methylisoxazole-4-carbohydrazide. J. Mol. Struct..

[55] Jin Y.-X., Zhong A.-G., Zhang Y., Pan F.-Y. (2011). Synthesis, crystal structure, spectroscopic properties, antibacterial activity and theoretical studies of a novel difunctional acylhydrazone. J. Mol. Struct..

[56] Rodríguez-Argüelles M.C., Cao R., García-Deibe A.M., Pelizzi C., Sanmartín-Matalobos J., Zani F. (2009). Antibacterial and antifungal activity of metal(II) complexes of acylhydrazones of 3-isatin and 3-(N-methyl)isatin. Polyhedron.

[57] Wang X.L., Zhang Y.B., Tang J.F., Yang Y.S., Chen R.Q., Zhang F., Zhu H.L. (2012). Design, synthesis and antibacterial activities of vanillic acylhydrazone derivatives as potential β-ketoacyl-acyl carrier protein synthase III (FabH) inhibitors. Eur. J. Med. Chem..

[58] Bonnett S.A., Dennison D., Files M., Bajpai A., Parish T. (2018). A class of hydrazones are active against non-replicating Mycobacterium tuberculosis. PLoS ONE.

[59] O’Dwyer K., Hackel M., Hightower S., Hoban D., Bouchillon S., Qin D., Aubart K., Zalacain M., Butler D. (2013). Comparative Analysis of the Antibacterial Activity of a Novel Peptide Deformylase Inhibitor, GSK1322322. Antimicrob. Agents Chemother..

[60] Guilherme F.D., Simonetti J.É., Folquitto L.R.S., Reis A.C.C., Oliver J.C., Dias A.L.T., Dias D.F., Carvalho D.T., Brandão G.C., de Souza T.B. (2019). Synthesis, chemical characterization and antimicrobial activity of new acylhydrazones derived from carbohydrates. J. Mol. Struct..

[61] Reis D., Despaigne A., Silva J., Silva N., Vilela C., Mendes I., Takahashi J., Beraldo H. (2013). Structural Studies and Investigation on the Activity of Imidazole-Derived Thiosemicarbazones and Hydrazones against Crop-Related Fungi. Molecules.

[62] Tian B., He M., Tang S., Hewlett I., Tan Z., Li J., Jin Y., Yang M. (2009). Synthesis and antiviral activities of novel acylhydrazone derivatives targeting HIV-1 capsid protein. Bioorganic Med. Chem. Lett..

[63] Wang Z., Xie D., Gan X., Zeng S., Zhang A., Yin L., Song B., Jin L., Hu D. (2017). Synthesis, antiviral activity, and molecular docking study of trans-ferulic acid derivatives containing acylhydrazone moiety. Bioorganic Med. Chem. Lett..

[64] Herschhorn A., Gu C., Espy N., Richard J., Finzi A., Sodroski J.G. (2014). A broad HIV-1 inhibitor blocks envelope glycoprotein transitions critical for entry. Nat. Chem. Biol..

[65] Yoneda J.D., Albuquerque M.G., Leal K.Z., Santos F.D.C., Batalha P.N., Brozeguini L., Seidl P.R., de Alencastro R.B., Cunha A.C., de Souza M.C.B.V. (2014). Docking of anti-HIV-1 oxoquinoline-acylhydrazone derivatives as potential HSV-1 DNA polymerase inhibitors. J. Mol. Struct..

[66] Reznichenko O., Quillévéré A., Martins R.P., Loaëc N., Kang H., Lista M.J., Beauvineau C., González-García J., Guillot R., Voisset C. (2019). Novel cationic bis(acylhydrazones) as modulators of Epstein–Barr virus immune evasion acting through disruption of interaction between nucleolin and G-quadruplexes of EBNA1 mRNA. Eur. J. Med. Chem..

[67] Zhao Z.X., Cheng L.P., Li M., Pang W., Wu F.H. (2019). Discovery of novel acylhydrazone neuraminidase inhibitors. Eur. J. Med. Chem..

[68] Abdel-Aal M.T., El-Sayed W.A., El-Ashry E.-S.H. (2006). Synthesis and Antiviral Evaluation of Some Sugar Arylglycinoylhydrazones and Their Oxadiazoline Derivatives. Arch. Pharm..

[69] Riccardi L., Genna V., De Vivo M. (2018). Metal–ligand interactions in drug design. Nat. Rev. Chem..

[70] Inam A., Siddiqui S.M., Macedo T.S., Moreira D.R.M., Leite A.C.L., Soares M.B.P., Azam A. (2014). Design, synthesis and biological evaluation of 3-[4-(7-chloro-quinolin-4-yl)-piperazin-1-yl]-propionic acid hydrazones as antiprotozoal agents. Eur. J. Med. Chem..

[71] Melnyk P., Leroux V., Sergheraert C., Grellier P. (2006). Design, synthesis and in vitro antimalarial activity of an acylhydrazone library. Bioorganic Med. Chem. Lett..

[72] dos Santos Filho J.M., de Queiroz e Silva D.M.A., Macedo T.S., Teixeira H.M.P., Moreira D.R.M., Challal S., Wolfender J.-L., Queiroz E.F., Soares M.B.P. (2016). Conjugation of N -acylhydrazone and 1,2,4-oxadiazole leads to the identification of active antimalarial agents. Bioorganic Med. Chem..

[73] Shakdofa M.M.E., Shtaiwi M.H., Morsy N., Abdel-rassel T.M.A. (2014). Metal complexes of hydrazones and their biological, analytical and catalytic applications: A review. Main Group Chem..

[74] dos Santos Filho J.M., Leite A.C.L., de Oliveira B.G., Moreira D.R.M., Lima M.S., Soares M.B.P., Leite L.F.C.C. (2009). Design, synthesis and cruzain docking of 3-(4-substituted-aryl)-1,2,4-oxadiazole-N-acylhydrazones as anti-Trypanosoma cruzi agents. Bioorganic Med. Chem..

[75] Jacomini A.P., Silva M.J.V., Silva R.G.M., Gonçalves D.S., Volpato H., Basso E.A., Paula F.R., Nakamura C.V., Sarragiotto M.H., Rosa F.A. (2016). Synthesis and evaluation against Leishmania amazonensis of novel pyrazolo[3,4-d]pyridazinone-*N*-acylhydrazone-(bi)thiophene hybrids. Eur. J. Med. Chem..

[76] Romeiro N.C., Aguirre G., Hernández P., González M., Cerecetto H., Aldana I., Pérez-Silanes S., Monge A., Barreiro E.J., Lima L.M. (2009). Synthesis, trypanocidal activity and docking studies of novel quinoxaline-N-acylhydrazones, designed as cruzain inhibitors candidates. Bioorganic Med. Chem..

[77] Bezerra-Netto H.J.C., Lacerda D.I., Miranda A.L.P., Alves H.M., Barreiro E.J., Fraga C.A.M. (2006). Design and synthesis of 3,4-methylenedioxy-6-nitrophenoxyacetylhydrazone derivatives obtained from natural safrole: New lead-agents with analgesic and antipyretic properties. Bioorganic Med. Chem..

[78] Duarte C.D., Tributino J.L.M., Lacerda D.I., Martins M.V., Alexandre-Moreira M.S., Dutra F., Bechara E.J.H., De-Paula F.S., Goulart M.O.F., Ferreira J. (2007). Synthesis, pharmacological evaluation and electrochemical studies of novel 6-nitro-3,4-methylenedioxyphenyl-N-acylhydrazone derivatives: Discovery of LASSBio-881, a new ligand of cannabinoid receptors. Bioorganic Med. Chem..

[79] Tributino J.L.M., Duarte C.D., Corrêa R.S., Doriguetto A.C., Ellena J., Romeiro N.C., Castro N.G., Miranda A.L.P., Barreiro E.J., Fraga C.A.M. (2009). Novel 6-methanesulfonamide-3,4-methylenedioxyphenyl-N-acylhydrazones: Orally effective anti-inflammatory drug candidates. Bioorganic Med. Chem..

[80] Hernández P., Cabrera M., Lavaggi M.L., Celano L., Tiscornia I., Rodrigues da Costa T., Thomson L., Bollati-Fogolín M., Miranda A.L.P., Lima L.M. (2012). Discovery of new orally effective analgesic and anti-inflammatory hybrid furoxanyl N-acylhydrazone derivatives. Bioorganic Med. Chem..

[81] da Silva Y.K.C., Augusto C.V., de Castro Barbosa M.L., de Albuquerque Melo G.M., de Queiroz A.C., de Lima Matos Freire Dias T., Júnior W.B., Barreiro E.J., Lima L.M., Alexandre-Moreira M.S. (2010). Synthesis and pharmacological evaluation of pyrazine N-acylhydrazone derivatives designed as novel analgesic and anti-inflammatory drug candidates. Bioorganic Med. Chem..

[82] Cerqueira J.V., Meira C.S., Santos E.d.S., de Aragão França L.S., Vasconcelos J.F., Nonaka C.K.V., de Melo T.L., dos Santos Filho J.M., Moreira D.R.M., Soares M.B.P. (2019). Anti-inflammatory activity of SintMed65, an N-acylhydrazone derivative, in a mouse model of allergic airway inflammation. Int. Immunopharmacol..

[83] Meira C.S., dos Santos Filho J.M., Sousa C.C., Anjos P.S., Cerqueira J.V., Dias Neto H.A., da Silveira R.G., Russo H.M., Wolfender J.-L., Queiroz E.F. (2018). Structural design, synthesis and substituent effect of hydrazone-N-acylhydrazones reveal potent immunomodulatory agents. Bioorganic Med. Chem..

[84] Moraes A.D.T.d.O., de Miranda M.D.S., Jacob Í.T.T., Amorim C.A.d.C., de Moura R.O., da Silva S.Â.S., Soares M.B.P., de Almeida S.M.V., Souza T.R.C.d.L., de Oliveira J.F. (2018). Synthesis, in vitro and in vivo biological evaluation, COX-1/2 inhibition and molecular docking study of indole-N-acylhydrazone derivatives. Bioorganic Med. Chem..

[85] Kümmerle A.E., Vieira M.M., Schmitt M., Miranda A.L.P., Fraga C.A.M., Bourguignon J.-J., Barreiro E.J. (2009). Design, synthesis and analgesic properties of novel conformationally-restricted N-acylhydrazones (NAH). Bioorganic Med. Chem. Lett..

[86] Guimarães E.T., dos Santos T.B., Silva D.K.C., Meira C.S., Moreira D.R.M., da Silva T.F., Salmon D., Barreiro E.J., Soares M.B.P. (2018). Potent immunosuppressive activity of a phosphodiesterase-4 inhibitor N-acylhydrazone in models of lipopolysaccharide-induced shock and delayed-type hypersensitivity reaction. Int. Immunopharmacol..

[87] Jakubek M., Kejík Z., Kaplánek R., Antonyová V., Hromádka R., Šandriková V., Sýkora D., Martásek P., Král V. (2019). Hydrazones as novel epigenetic modulators: Correlation between TET 1 protein inhibition activity and their iron(II) binding ability. Bioorganic Chem..

[88] Özturan Özer E., Unsal Tan O., Ozadali K., Küçükkılınç T., Balkan A., Uçar G. (2013). Synthesis, molecular modeling and evaluation of novel N′-2-(4-benzylpiperidin-/piperazin-1-yl)acylhydrazone derivatives as dual inhibitors for cholinesterases and Aβ aggregation. Bioorganic Med. Chem. Lett..

[89] Dias Viegas F.P., de Freitas Silva M., Divino da Rocha M., Castelli M.R., Riquiel M.M., Machado R.P., Vaz S.M., Simões de Lima L.M., Mancini K.C., Marques de Oliveira P.C. (2018). Design, synthesis and pharmacological evaluation of N -benzyl-piperidinyl-aryl-acylhydrazone derivatives as donepezil hybrids: Discovery of novel multi-target anti-alzheimer prototype drug candidates. Eur. J. Med. Chem..

[90] Canal-Martín A., Sastre J., Sánchez-Barrena M.J., Canales A., Baldominos S., Pascual N., Martínez-González L., Molero D., Fernández-Valle M.E., Sáez E. (2019). Insights into real-time chemical processes in a calcium sensor protein-directed dynamic library. Nat. Commun..

[91] Zhang F., Wang X.-L., Shi J., Wang S.-F., Yin Y., Yang Y.-S., Zhang W.-M., Zhu H.-L. (2014). Synthesis, molecular modeling and biological evaluation of N-benzylidene-2-((5-(pyridin-4-yl)-1,3,4-oxadiazol-2-yl)thio)acetohydrazide derivatives as potential anticancer agents. Bioorganic Med. Chem..

[92] Gage J.L., Onrust R., Johnston D., Osnowski A., MacDonald W., Mitchell L., Ürögdi L., Rohde A., Harbol K., Gragerov S. (2011). N-Acylhydrazones as inhibitors of PDE10A. Bioorganic Med. Chem. Lett..

[93] He J.-B., Feng L.-L., Li J., Tao R.-J., Ren Y.-L., Wan J., He H.-W. (2014). Design, synthesis and molecular modeling of novel N-acylhydrazone derivatives as pyruvate dehydrogenase complex E1 inhibitors. Bioorganic Med. Chem..

[94] Gao G.-R., Liu J.-L., Mei D.-S., Ding J., Meng L.-H., Duan W.-H. (2015). Design, synthesis and biological evaluation of acylhydrazone derivatives as PI3K inhibitors. Chin. Chem. Lett..

[95] van der Vlag R., Guo H., Hapko U., Eleftheriadis N., Monjas L., Dekker F.J., Hirsch A.K.H. (2019). A combinatorial approach for the discovery of drug-like inhibitors of 15-lipoxygenase-1. Eur. J. Med. Chem..

[96] Rupiani S., Buonfiglio R., Manerba M., Di Ianni L., Vettraino M., Giacomini E., Masetti M., Falchi F., Di Stefano G., Roberti M. (2015). Identification of N-acylhydrazone derivatives as novel lactate dehydrogenase A inhibitors. Eur. J. Med. Chem..

[97] Ünsal-Tan O., Özden K., Rauk A., Balkan A. (2010). Synthesis and cyclooxygenase inhibitory activities of some N-acylhydrazone derivatives of isoxazolo[4,5-d]pyridazin-4(5H)-ones. Eur. J. Med. Chem..

[98] Carcelli M., Rogolino D., Gatti A., De Luca L., Sechi M., Kumar G., White S.W., Stevaert A., Naesens L. (2016). N-acylhydrazone inhibitors of influenza virus PA endonuclease with versatile metal binding modes. Sci. Rep..

[99] Parlar S., Sayar G., Tarikogullari A.H., Karadagli S.S., Alptuzun V., Erciyas E., Holzgrabe U. (2019). Synthesis, bioactivity and molecular modeling studies on potential anti-Alzheimer piperidinehydrazide-hydrazones. Bioorganic Chem..

[100] Anastassova N.O., Yancheva D.Y., Mavrova A.T., Kondeva-Burdina M.S., Tzankova V.I., Hristova-Avakumova N.G., Hadjimitova V.A. (2018). Design, synthesis, antioxidant properties and mechanism of action of new N,N′-disubstituted benzimidazole-2-thione hydrazone derivatives. J. Mol. Struct..

[101] Socea L.I., Visan D.C., Barbuceanu S.F., Apostol T.V., Bratu O.G., Socea B. (2018). The Antioxidant Activity of Some Acylhydrazones with Dibenzo[a,d][7]annulene Moiety. Rev. Chim..

[102] Costa D.G., da Silva J.S., Kümmerle A.E., Sudo R.T., Landgraf S.S., Caruso-Neves C., Fraga C.A.M., de Lacerda Barreiro E.J., Zapata-Sudo G. (2010). LASSBio-294, A Compound With Inotropic and Lusitropic Activity, Decreases Cardiac Remodeling and Improves Ca^2+^ Influx Into Sarcoplasmic Reticulum After Myocardial Infarction. Am. J. Hypertens..

[103] da Silva J.S., Pereira S.L., Maia R.D.C., Landgraf S.S., Caruso-Neves C., Kümmerle A.E., Fraga C.A.M., Barreiro E.J., Sudo R.T., Zapata-Sudo G. (2014). N-acylhydrazone improves exercise intolerance in rats submitted to myocardial infarction by the recovery of calcium homeostasis in skeletal muscle. Life Sci..

[104] Zapata-Sudo G., Pereira S.L., Beiral H.J.V., Kummerle A.E., Raimundo J.M., Antunes F., Sudo R.T., Barreiro E.J., Fraga C.A.M. (2010). Pharmacological Characterization of (3-Thienylidene)-3,4-Methylenedioxybenzoylhydrazide: A Novel Muscarinic Agonist With Antihypertensive Profile. Am. J. Hypertens..

[105] Sathler P.C., Lourenço A.L., Rodrigues C.R., da Silva L.C.R.P., Cabral L.M., Jordão A.K., Cunha A.C., Vieira M.C.B., Ferreira V.F., Carvalho-Pinto C.E. (2014). In vitro and in vivo analysis of the antithrombotic and toxicological profile of new antiplatelets N-acylhydrazone derivatives and development of nanosystems. Thromb. Res..

[106] Lima L.M., Frattani F.S., dos Santos J.L., Castro H.C., Fraga C.A.M., Zingali R.B., Barreiro E.J. (2008). Synthesis and anti-platelet activity of novel arylsulfonate–acylhydrazone derivatives, designed as antithrombotic candidates. Eur. J. Med. Chem..

[107] Jordão A.K., Ferreira V.F., Lima E.S., de Souza M.C.B.V., Carlos E.C.L., Castro H.C., Geraldo R.B., Rodrigues C.R., Almeida M.C.B., Cunha A.C. (2009). Synthesis, antiplatelet and in silico evaluations of novel N-substituted-phenylamino-5-methyl-1H-1,2,3-triazole-4-carbohydrazides. Bioorganic Med. Chem..

[108] Alencar A.K.N., Pereira S.L., da Silva F.E., Mendes L.V.P., Cunha V.D.M.N., Lima L.M., Montagnoli T.L., Caruso-Neves C., Ferraz E.B., Tesch R. (2014). N-acylhydrazone derivative ameliorates monocrotaline-induced pulmonary hypertension through the modulation of adenosine AA2R activity. Int. J. Cardiol..

[109] Silva A.G., Zapata-Sudo G., Kummerle A.E., Fraga C.A.M., Barreiro E.J., Sudo R.T. (2005). Synthesis and vasodilatory activity of new N-acylhydrazone derivatives, designed as LASSBio-294 analogues. Bioorganic Med. Chem..

[110] Kümmerle A.E., Raimundo J.M., Leal C.M., da Silva G.S., Balliano T.L., Pereira M.A., de Simone C.A., Sudo R.T., Zapata-Sudo G., Fraga C.A.M. (2009). Studies towards the identification of putative bioactive conformation of potent vasodilator arylidene N-acylhydrazone derivatives. Eur. J. Med. Chem..

[111] Feng H., Li C., Shu S., Liu H., Zhang H. (2019). A11, a novel diaryl acylhydrazone derivative, exerts neuroprotection against ischemic injury in vitro and in vivo. Acta Pharmacol. Sin..

[112] Vilková M., Hudáčová M., Palušeková N., Jendželovský R., Almáši M., Béres T., Fedoročko P., Kožurková M. (2022). Acridine Based N-Acylhydrazone Derivatives as Potential Anticancer Agents: Synthesis, Characterization and ctDNA/HSA Spectroscopic Binding Properties. Molecules.

[113] Banumathi, Sherapura A., Malojirao V.H., Zabiulla, Sharath B.S., Thirusangu P., Mahmood R., Kumari N.S., Baliga S.M., Khanum S.A. (2022). Antiproliferative pharmacophore azo-hydrazone analogue BT-1F exerts death signalling pathway targeting STAT3 in solid tumour. Pharmacol. Rep..

[114] Osmaniye D., Levent S., Karaduman A., Ilgın S., Özkay Y., Kaplancıklı Z. (2018). Synthesis of New Benzothiazole Acylhydrazones as Anticancer Agents. Molecules.

[115] Biagi G.L., Guerra M.C., Barbaro A.M., Gamba M.F. (1970). Influence of lipophilic character on the antibacterial activity of cephalosporins and penicillins. J. Med. Chem..

[116] Ebejer J.-P., Charlton M.H., Finn P.W. (2016). Are the physicochemical properties of antibacterial compounds really different from other drugs?. J. Cheminformatics.

[117] Zitko J., Doležal M. (2016). Enoyl acyl carrier protein reductase inhibitors: An updated patent review (2011–2015). Expert Opin. Ther. Pat..

[118] Shah M.A., Uddin A., Shah M.R., Ali I., Ullah R., Hannan P.A., Hussain H. (2022). Synthesis and Characterization of Novel Hydrazone Derivatives of Isonicotinic Hydrazide and Their Evaluation for Antibacterial and Cytotoxic Potential. Molecules.

[119] Yao X., Hu H., Wang S., Zhao W., Song M., Zhou Q. (2021). Synthesis, Antimicrobial Activity, and Molecular Docking Studies of Aminoguanidine Derivatives Containing an Acylhydrazone Moiety. Iran. J. Pharm. Res..

[120] Kumar P., Kadyan K., Duhan M., Sindhu J., Singh V., Saharan B.S. (2017). Design, synthesis, conformational and molecular docking study of some novel acyl hydrazone based molecular hybrids as antimalarial and antimicrobial agents. Chem. Cent. J..

[121] Coimbra E.S., Nora de Souza M.V., Terror M.S., Pinheiro A.C., da Trindade Granato J. (2019). Synthesis, biological activity, and mechanism of action of new 2-pyrimidinyl hydrazone and N-acylhydrazone derivatives, a potent and new classes of antileishmanial agents. Eur. J. Med. Chem..

[122] Tejera E., Munteanu C.R., López-Cortés A., Cabrera-Andrade A., Pérez-Castillo Y. (2020). Drugs Repurposing Using QSAR, Docking and Molecular Dynamics for Possible Inhibitors of the SARS-CoV-2 Mpro Protease. Molecules.

[123] Cordeiro N.d.M., Freitas R.H.C.N., Fraga C.A.M., Fernandes P.D. (2020). New 2-amino-pyridinyl-N-acylhydrazones: Synthesis and identification of their mechanism of anti-inflammatory action. Biomed. Pharmacother..

[124] da Silva Monteiro C.E., Franco Á.X., Sousa J.A.O., Matos V.E.A., de Souza E.P., Fraga C.A.M., Barreiro E.J., de Souza M.H.L.P., Soares P.M.G., Barbosa A.L.R. (2019). Gastroprotective effects of N-acylarylhydrazone derivatives on ethanol-induced gastric lesions in mice are dependent on the NO/cGMP/KATP pathway. Biochem. Pharmacol..

